# Optical sorting: past, present and future

**DOI:** 10.1038/s41377-024-01734-5

**Published:** 2025-02-27

**Authors:** Meng Yang, Yuzhi Shi, Qinghua Song, Zeyong Wei, Xiong Dun, Zhiming Wang, Zhanshan Wang, Cheng-Wei Qiu, Hui Zhang, Xinbin Cheng

**Affiliations:** 1 Institute of Precision Optical Engineering, School of Physics Science and Engineering, Tongji University, Shanghai, 200092 China; 2 MOE Key Laboratory of Advanced Micro-Structured Materials, Shanghai, 200092 China; 3 Shanghai Institute of Intelligent Science and Technology, Tongji University, Shanghai, 200092 China; 4 Shanghai Frontiers Science Center of Digital Optics, Shanghai, 200092 China; 5 Tsinghua Shenzhen International Graduate School, Tsinghua University, Shenzhen, 518055 China; 6 Institute of Fundamental and Frontier Sciences, University of Electronic Science and Technology of China, Chengdu, 610054 China; 7 Department of Electrical and Computer Engineering, National University of Singapore, Singapore, 117583 Singapore

**Keywords:** Optical manipulation and tweezers, Nanophotonics and plasmonics

## Abstract

Optical sorting combines optical tweezers with diverse techniques, including optical spectrum, artificial intelligence (AI) and immunoassay, to endow unprecedented capabilities in particle sorting. In comparison to other methods such as microfluidics, acoustics and electrophoresis, optical sorting offers appreciable advantages in nanoscale precision, high resolution, non-invasiveness, and is becoming increasingly indispensable in fields of biophysics, chemistry, and materials science. This review aims to offer a comprehensive overview of the history, development, and perspectives of various optical sorting techniques, categorised as *passive* and *active* sorting methods. To begin, we elucidate the fundamental physics and attributes of both conventional and exotic optical forces. We then explore sorting capabilities of active optical sorting, which fuses optical tweezers with a diversity of techniques, including Raman spectroscopy and machine learning. Afterwards, we reveal the essential roles played by deterministic light fields, configured with lens systems or metasurfaces, in the passive sorting of particles based on their varying sizes and shapes, sorting resolutions and speeds. We conclude with our vision of the most promising and futuristic directions, including AI-facilitated ultrafast and bio-morphology-selective sorting. It can be envisioned that optical sorting will inevitably become a revolutionary tool in scientific research and practical biomedical applications.

## Abstract (web summary)

This review offers a comprehensive overview of the history, development, and perspectives of various optical sorting techniques, categorised as *passive* and *active* sorting methods.

## Graphical abstract

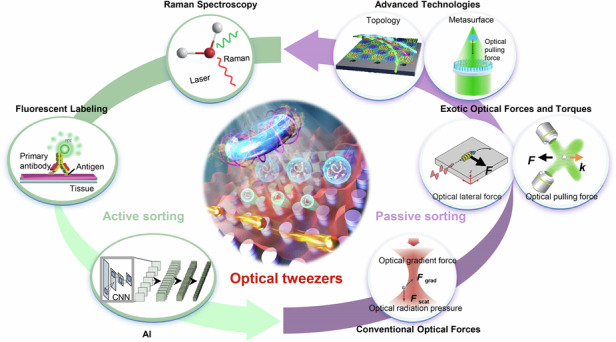

## Introduction

Size, shape and refractive index are among the intrinsic characteristics of particles, which determine their unique properties in various scientific fields such as physics, chemistry, mechanics, biomedicine, energy, and environmental sciences^1–5^. Essentially, particle size can have a significant impact on the melting point^6^, effective refractive index^7^, and other physical properties of nanomaterials. For example, nanoparticles exhibit physical properties that are drastically distinct from macroparticles, such as quantum size effects^8^. Small particles have greater surface-volume ratios, allowing more reactants to contact the surface of the particle, thus accelerating the speeds of chemical reactions^9,10^. This size effect is of exceeding importance in chemocatalysis^11^ and drug release^12^.

In optics, metallic nanoparticles may excite the surface plasmon resonance (SPR), thus profoundly enhancing light absorption and scattering. Mie-resonant dielectric nanoparticles with certain sizes can induce multipoles, toroidal dipoles, and sometimes bound states in the continuum (BICs)^13–16^, manoeuvring electromagnetic waves in unconventional and efficient ways. In biology, exosomes^17–19^ with different sizes behave distinctively in composition, biological function, disease diagnosis, potential applications, etc. In drug delivery systems, controlling particle sizes of drug carriers can improve drug biocompatibility, and prolong circulation time in the body, consequently enhancing therapeutic effects^20,21^. In energy sciences, for instance, in batteries and other energy storage materials, optimizing particle size can improve the ion transport rate and charge storage capacity of electrode materials, thereby enhancing energy conversion and storage efficiency^22–24^.

Albeit their significant importance, nanoparticles, especially bioparticles, may display a wide range of size and shape distributions during the synthesis and culturing processes^25–30^. Thus, sorting them with a high purity becomes an open question in diverse disciplines. Among a plethora of approaches, acoustics, dielectrophoresis, deterministic lateral displacement, membrane (micro/nanopores) and optical tweezers stand out as prevalent and profound methods, giving rise to rich applications in particle sorting^7,31–33^. Each approach has its own advantages and disadvantages, warranting extensive discussions. However, delving into these details is outside the scope of this article.

In this paper, we concentrate on an archetypal sorting method—optical sorting—which integrates optical tweezers with various auxiliary techniques including microfluidics, artificial intelligence (AI), imaging processes, immunoassays, and more. Optical sorting inherits unequivocal advantages from optical tweezers, such as non-invasiveness, small size, high resolution, etc. Typical optical forces in optical tweezers include conventional optical gradient force (OGF) and optical radiation pressure (ORP), while recently emerged exotic optical forces are the optical pulling force (OPF) and optical lateral force (OLF). Each of these forces can be harnessed for optical sorting, leveraging their distinct characteristics. There are ample papers that review comprehensively optical tweezers^34–38^, highlighting the great potential of this technique in particle sorting. However, there is a lack of an overview that comprehensively summarizes the underlying physics and recent advances in this field.

**Fig. 1:**
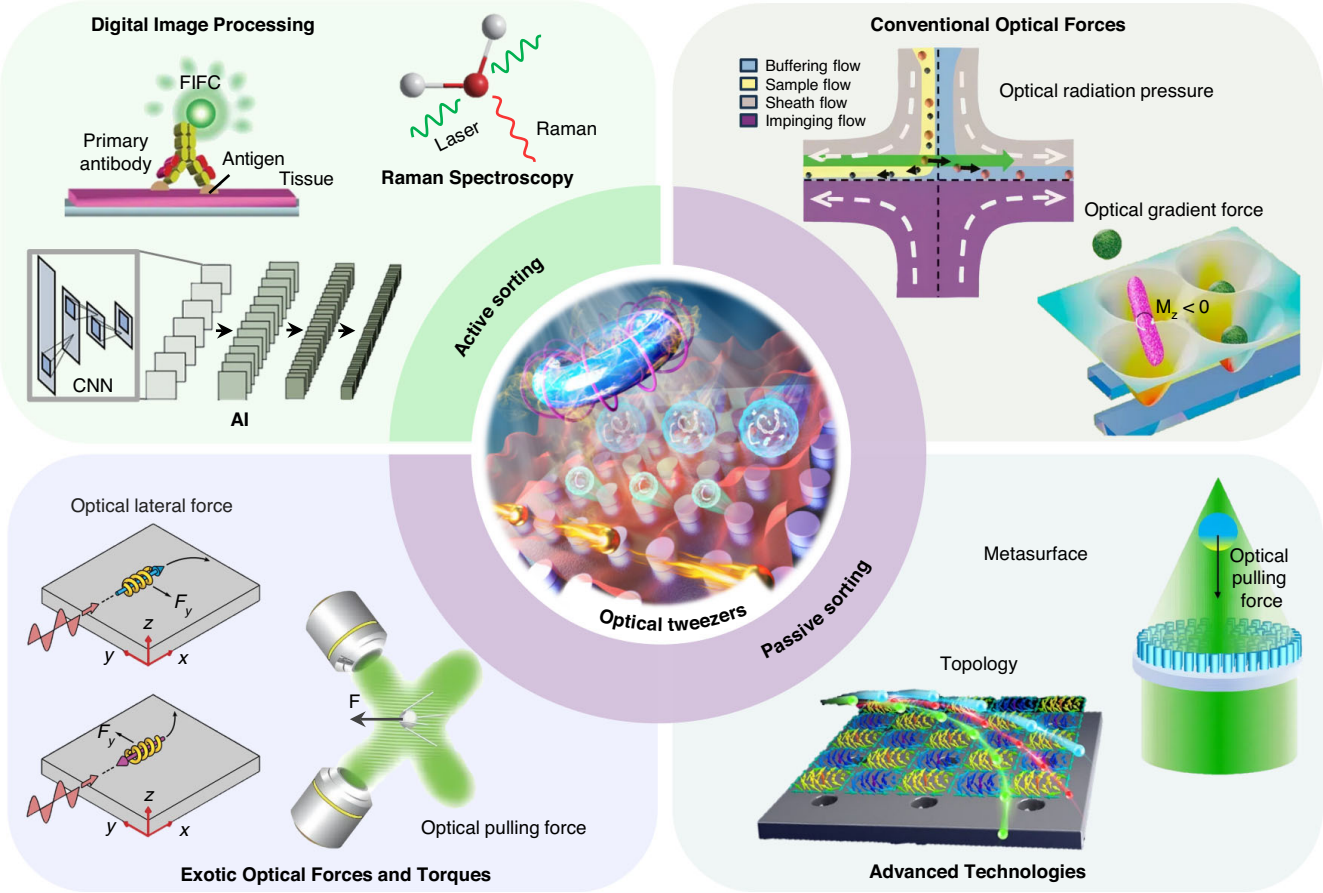
Overview of principles of particle sorting using optical tweezers, which can be categorized as active sorting and passive sorting Active sorting techniques involve particle pre-processing, such as Raman spectroscopy. Reproduced with permission^548^. Copyright 2022, Springer Nature; fluorescent labelling. Reproduced with permission^240^. Copyright 2022, Springer Nature; and AI algorithms. Reproduced with permission^549^. Copyright 2021, Springer Nature. Passive sorting methods involve conventional optical forces (such as ORP. Reproduced with permission^297^. Copyright 2016, American Chemical Society; OGF. Reproduced with permission^372^. Copyright 2019, American Chemical Society and so on), exotic optical forces and torques (such as the OPF. Reproduced with permission^66^. Copyright 2013, Springer Nature); and the OLF. Reproduced with permission^69^. Copyright 2014, Springer Nature, as well as advanced technologies like the Metasurface. Reproduced with permission^341^. Copyright 2023, Chinese Physical Society and IOP Publishing Ltd; and Topological approaches. Reproduced with permission^131^. Copyright 2023, American Chemical Society

We start with a quick glimpse of this field, which can be categorized as passive and active sorting, as shown in Fig. 1. Active sorting entails the use of mobile and adjustable optical tweezers, enabling dynamic control of particle movement through external signals from different particles, which can be fluorescence light, Raman signals, data from machine learning, etc. Passive sorting is the most commonly investigated and implemented using various approaches. For example, optical sorting can be achieved through the use of the ORP and OGF, which have been extensively studied over the past three decades. The last decade has also witnessed burgeoning developments of exotic optical forces, such as the intriguing OPF, OLF, inverse optical torque, etc. Recently, metasurfaces have prevailed as powerful paradigms for optical manipulation and sorting as they are exceedingly efficient in steering electromagnetic fields^39–43^. Notably, the light field can further be enhanced using multipoles and topology, thereby significantly increasing the resolution of optical sorting.

After a short retrospect, we then discuss thoroughly details of various active and passive optical sorting techniques, covering the underlying physics, practical applications, as well as their respective advantages and disadvantages. Finally, this article concludes with our prospect of how optical sorting can be further developed to facilitate a broader range of biological and clinical applications, as well as support advancements in physical and chemical studies.

## A short view of the roadmap for optical sorting

**Fig. 2:**
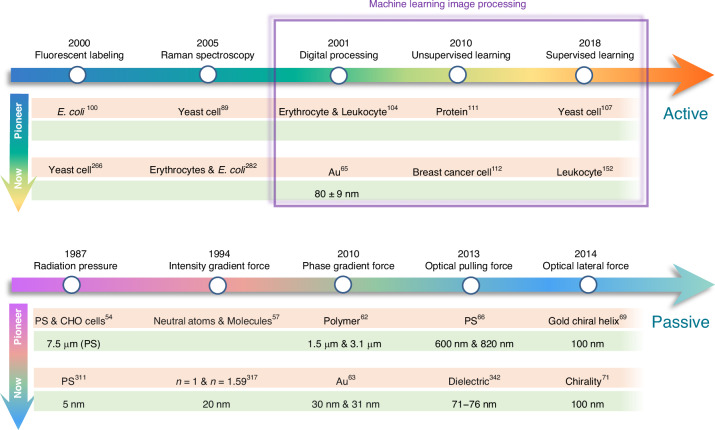
Roadmap on optical sorting. The upper and lower right arrows signify milestones of each principle employed in active and passive sorting techniques, respectively. The arrows on the left depict advances in optical sorting at the inception of the technology (marked “Pioneer”) and the current sorting limitations (marked “Now”)

Optical tweezers were invented by Prof. Ashkin during the 1970s and 1980s. Initially, two typical optical forces are proposed and extensively investigated, which are the ORP^44^ and OGF^45^. The ORP originates from the momentum transfer of propagating photons and can be utilized to propel particles. Typical applications include the solar sail and optical levitation^46–50^. With the assistance of energy level conversion of the atom^51–53^, atom cooling was later proposed and subsequently awarded the Nobel Prize in Physics in 1997. Optical sorting by the ORP was conducted by Buican et al. to sort Chinese Hamster Ovary cells based on different optical forces acting on them^54^, as shown in Fig. 2. This sorting technique, based on radiation pressure, is later well known as “optical chromatography”^55^, has been widely used to separate a wide variety of biological and artificial particles, including bacteria, cells, metallic, and dielectric nanoparticles^7,56^.

The OGF captures particles towards the regions of higher intensity when the light field has an intensity gradient^45^. This force is the most widely used in optical tweezers and was used for sorting neutral atoms and molecules in 1994^57^. After 30 years of development, this force can now be used to sort sub-100 nm proteins^58^ and viruses^59^. Interestingly, leveraging the Fano enhancement, Cao and Qiu theoretically demonstrated the sorting of chiral nanoparticles as small as 20 nm using the chiral OGF^60^.

The phase-gradient force is a type of force that arises from the gradient of phase. It was first systematically formulated and investigated by Roichman et al. in 2008^61^ and used for sorting polymers with sizes of 1.5 µm and 3.1 µm in 2010^62^. Recently, this force has been deployed to sort sub-100 nm metallic nanoparticles with a resolution of sub-10 nm^63–65^. Exotic optical forces, such as the OPF and OLF, are promising candidates for precise optical sorting, particularly for special particles like core-shell and chiral particles. The first experimental demonstration of the OPF was carried out in 2013 by Brzobohatý et al., who revealed the capability of this force in sorting particles with high precision, e.g., sorting 600-nm and 820-nm polystyrene particles^66^. Recognizing the potential, numerous studies have emerged, achieving a sorting size as small as 130 nm for silicon nanoparticles^67^, while another work has envisioned a sorting size of 78 nm for silica nanoparticles^68^. The OLF was proposed in 2014 by two groups independently^69,70^. Wang and Chan demonstrated the sorting of chiral helix with different handedness and size from 60 to 100 nm^69^. Ying proposed longitudinal polarization vortex structures to sort 100 nm enantiomers^71^. Due to the relatively small magnitude of the OLF, experimental studies typically focus on handling microparticles^72^.

**Fig. 3:**
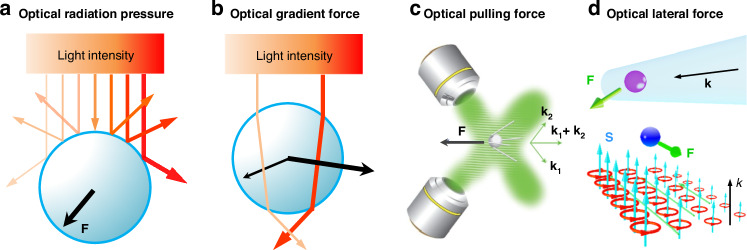
Schematic of four types of optical forces. **a** optical radiation pressure (ORP). It can arise from the light scattering/reflection by the particle. **b** optical gradient force (OGF). It can be easily comprehended by the light refraction when the particle is in the ray-optics regime. **c** Optical pulling force (OPF). This force can occur on a microparticle in a two-wave-interference-induced Bessel beam. Reproduced with permission^66^. Copyright 2013, Springer Nature. **d** Optical lateral force (OLF). The inhomogeneity of the spin angular momentum generates the transverse Belinfante spin momentum and OLF

The illustration of four typical optical forces is shown in Fig. 3. The ORP occurs ubiquitously when light is scattered by the particle, as shown in Fig. 3a. When the particle is in the dipole range (radius *a* $\ll$ wavelength *λ*), the ORP aligns with the direction of the wavevector^73–75^. It can be comprehended through the momentum exchange induced by the ray reflection when the particle is in the ray-optics regime (radius *a* $\gg$ wavelength *λ*)^76^. The most commonly used OGF arises from the gradient of light intensity^77^, as depicted in Fig. 3b. In the dipole theory, it is understood from that a small particle with a polarized dipole moment is subjected to a field with an electric gradient^78,79^. It is easily observed through ray refraction after passing through a large particle in the ray-optics regime^80^. The counterintuitive OPF^73,81–83^, which acts in the opposite direction of the wave vector, does not necessarily occur under conditions of a negative Poynting vector. Instead, it is often accompanied by dominant forward scattering induced by special particles, light waves, or both^37,73,84^, as illustrated in Fig. 3c. The latest emerged OLF can arise from a variety of mechanisms^35^, for instance, the renowned Belinfante spin momentum^70,85–87^, as depicted in Fig. 3d.

Raman spectroscopy, known as a “molecular fingerprint” for substance identification, was first discovered in 1928 by the Indian physicist Sir Chandrasekhara Venkata Raman^88^. In 2005, it was integrated with optical tweezers to differentiate and separate live and dead yeast cells^89^, showcasing its effectiveness as a robust label-free technique for particle identification.

The fluorescent staining technique is a method used in biology and biotechnology to visualize and identify specific molecules or structures within cells or tissues^90–92^. It is utilized to selectively label cell membranes^93,94^ or cell organelles^95–98^ through dye staining methods or the specific binding of antigens and antibodies^90,99^, causing them to emit light when excited by a specific wavelength of light. This method was integrated with optical tweezers in 2000 to sort *E. coli* and evaluate the single-cell viability^100^. In recent years, optical tweezers, combined with fluorescent staining techniques and Raman spectroscopy, have been utilized to sort a diverse range of cells, particles, and other entities, leveraging their capacity for accurate substance identification^101–103^.

Image processing methods are emerging as a prominent technique that is gradually being integrated into optical sorting processes. For instance, in 2001, a conventional image-processing system using threshold segmentation, background subtraction and edge-enhancement algorithms was used to identify single cells and then to sort out red blood cells from human peripheral blood using a dual-beam trap^104^. Utilizing threshold segmentation methods in digital image processing (DIP), particle edges can be discerned based on fluorescence intensity to distinguish particle sizes and facilitate particle sorting^105^. Moreover, by employing an intensity-phase beam shaping algorithm, nanoparticles can be manipulated according to their distinct positions and scattering intensities, thereby enabling the sorting of both gold (Au) and silver (Ag) nanoparticles^65^. Although traditional DIP methods have been widely used and have achieved some effectiveness in cell sorting, fundamentally, these methods lack adaptability to evolving conditions and often require manual intervention for new scenarios. With the development of AI algorithms such as machine learning^106^, data-driven machine learning image processing methods start to be applied to cell sorting and are generally categorized into supervised and unsupervised learning. In the supervised learning category, Yu et al. used optical imaging and k-nearest neighbor (KNN) algorithm to identify yeast cells and separate them in a microfluidic system in 2018^107^. Apart from that, classic examples include using Support Vector Machines (SVMs) to classify human chromosomes^108^ or using multilayer perceptrons (MLPs) to differentiate healthy and diseased cells based on mechanical properties^109^. KNN has also been successfully used to identify components of epilepsy^110^. Considering the difficulty in obtaining large labelled datasets, unsupervised learning has become effective, where patterns are inferred from input data rather than explicitly trained with labelled output. In 2010, a random forest model was utilized to differentiate proteins found in organelles and cell membranes^111^. The random forest method has also been used to classify breast cancer and normal single cells^112^. The flexibility and adaptability of machine learning rely on the dependency on large datasets, and the decision-making process lacks interpretability. In practice, adopting a collaborative approach that combines traditional image processing with the data-driven power of machine learning will be a promising pathway to achieve comprehensive and robust image analysis^113^. Here, a broad review of machine learning techniques in various cell sorting methods, including but not limited to optical sorting, has been conducted. These advanced machine learning image processing techniques have demonstrated their efficiency and crucial role in cell discrimination, thereby facilitating cell sorting, and can be seamlessly applied to optical sorting.

This article provides an overview of the historical developments, recent advancements, and prospects of optical sorting. For comparison, Table 1 summarizes the resolutions and limitations of various active and passive sorting techniques. The structure of this paper unfolds as follows:

**Table 1:** Summary of resolutions and limits of various active and passive sorting techniques

Sorting method	Technology	Particle size (*d*)	Particle types	Precision	Simulation(S) or Experiment(E)	Ref.
Active sorting	Algorithm	80 ± 9 nm	Au	18 nm	E	^65^
	Fluorescent labeling	A variety of cells or particles		-	E	^266^
	Raman spectroscopy	A variety of cells or particles		-	E	^282^
Passive sorting	Optical radiation pressure	5 nm	polystyrene	1 nm	S	^311^
	Optical gradient force	20 nm	*n* = 1 & *n* = 1.59	-	E	^317^
	Optical phase-gradient force	30–50 nm	Au	1 nm	S	^63^
	Optical pulling force	71–76 nm	dielectric	5 nm	S	^342^
	Optical lateral force	0.6–2 µm	chirality	~10 nm	E	^72^
	Potential well	10 nm & 20 nm	polystyrene	10 nm	S	^369^
	Holographic technique	1 µm & 1.9 µm	Silica	0.9 µm	E	^327^
	Optical field movement	204 nm & 310 nm	polystyrene	53 nm	E	^381^
	Optical field enhancement	10 nm & 20 nm	dielectric	10 nm	S	^427^
	Metasurface	4.2–5.4 nm	polystyrene	1.2 nm	S	^341^
	Topological field	90–120 nm	Au	10 nm	S	^131^

In the section “Active optical sorting”, we discuss several notable examples of active optical sorting, such as Raman spectroscopy and DIP. Especially, the DIP optical sorting is becoming more and more prominent due to the fast iteration of the machine learning algorithms. In the section “passive sorting”, we focus on a variety of passive optical sorting techniques, including sorting by the conventional ORP and OGF, by exotic optical forces such as the phase-gradient force, OPF and OLF. We also examine the impact of potential well control on the particle sorting, including variations in depth and shifts in position. Subsequently, we reveal a range of force enhancement mechanisms that can significantly push the boundaries of sorting size and speed. Notably, the force enhancement can also be realized by two typical configurations, i.e., metasurface and topology. Finally, we provide a brief summary and our own vision of this evolving yet enigmatic and promising field. It is anticipated that recent advancements in deep learning algorithms could inject new vigour and vitality into optical sorting, resulting in faster, higher precision, and improved specimen performances. This, in turn, will empower various scientific and clinical applications.

## Optical force

### Conventional optical forces

When subjected to a lightwave, a particle undergoes optical forces due to the interaction between light and matter, laying the groundwork for optical manipulation. Since there are numerous review papers available that consolidate the theory of optical forces^34,35,73,114–116^, we will now focus on outlining the most pertinent formulas related to optical sorting. For a dipole particle (radius *a* $\ll$ wavelength *λ*) with a permittivity *ε*_*p*_ and permeability *µ*_*p*_, the time-averaged optical force from an electromagnetic wavefield **E,** **B** is given as

1 $$\begin{array}{ll}{\bf{F}}={{\bf{F}}}_{e}+{{\bf{F}}}_{m}+{{\bf{F}}}_{em}\\ \qquad=\frac{1}{2}\mathrm{Re}\left\{{\underbrace{{\bf{p}}\cdot (\nabla){{\bf{E}}}^{\ast}}_{\sim {(ka)}^{3}}+\underbrace{{\bf{m}}\cdot (\nabla){{\bf{H}}}^{\ast}}_{\sim {(ka)}^{5}\,{\rm{for}}\,{\mu}_{{\rm{p}}}=\mu}-\underbrace{\frac{c{k}^{4}}{6\pi \sqrt{\varepsilon \mu}}({\bf{p}}\times {{\bf{m}}}^{\ast})}_{\sim{(ka)}^{8}\,{\rm{for}}\,{\mu}_{{\rm{p}}}=\mu}}\right\}\end{array}$$

where *k* is the wavenumber of light, *ε* and *µ* are the permittivity and permeability of the medium, respectively; ${{\bf{F}}}_{e}$, ${{\bf{F}}}_{m}$ and ${{\bf{F}}}_{em}$ represent the optical force on the induced electric dipole, magnetic dipole and electric-magnetic dipole interaction, respectively. The electric and magnetic dipole moments for an achiral particle can be expressed as ${\bf{p}}={\alpha }_{e}{\bf{E}}$ and ${\bf{m}}={\alpha }_{m}{\bf{H}}$, respectively, where *α*_*e*_ and *α*_*m*_ are the electric and magnetic polarizabilities for spherical dipolar particles. The polarizabilities can be obtained via the electric and magnetic first Mie coefficients *a*_1_^(1)^ and *b*_1_^(1)^, respectively, as

2 $$\begin{array}{c}{\alpha }_{e}=\frac{i6\pi \varepsilon }{{k}^{3}}{a}_{1}^{(1)}=\frac{{\alpha }_{e}^{0}}{1-\frac{{\varepsilon }_{p}-\varepsilon }{{\varepsilon }_{p}+\,2\varepsilon }[{(ka)}^{2}\,+\,\frac{2i}{3}{(ka)}^{3}]}\approx \frac{{\alpha }_{e}^{0}}{1-\frac{i{k}^{3}{\alpha }_{e}^{0}}{6\pi \varepsilon }}\\ {\alpha }_{m}=\frac{i6\pi }{\mu {k}^{3}}{b}_{1}^{(1)}=\frac{{\alpha }_{m}^{0}}{1-\frac{{\mu }_{p}-\mu }{{\mu }_{p}+2\mu }[{(ka)}^{2}\,+\,\frac{2i}{3}{(ka)}^{3}]}\approx \frac{{\alpha }_{m}^{0}}{1-\frac{i\mu {k}^{3}{\alpha }_{e}^{0}}{6\pi }}\end{array}$$

where ${\alpha }_{e}^{0}=4\pi \varepsilon {a}^{3}\frac{{\varepsilon }_{p}-\varepsilon }{{\varepsilon }_{p}+2\varepsilon }$ and ${\alpha }_{m}^{0}=4\pi {\mu }^{-1}{a}^{3}\frac{{\mu }_{p}-\mu }{{\mu }_{p}+2\mu }+O({[ka]}^{5})$ are quasistatic limits.

For a most common scenario that the dipole particle is non-magnetic, the optical force may be equal to the electric part of Eq. (1), which can be written as

3 $$\begin{array}{ll}{{\bf{F}}}_{e}=\frac{1}{4}\mathrm{Re}({\alpha }_{e})\nabla {|{\bf{E}}|}^{2}+\frac{2\omega }{\varepsilon }\text{Im}({\alpha }_{e}){{\bf{p}}}_{e}^{O}\\ \qquad\,\,=\frac{\mathrm{Re}({\alpha }_{e})}{4}\nabla {|{\bf{E}}|}^{2}+\frac{{\sigma }_{{\rm{ext}}}{n}_{m}}{c}{\bf{P}}-\frac{{\sigma }_{{\rm{ext}}}c}{2{n}_{m}}\nabla \times {{\bf{S}}}_{e}\\ \qquad\,\,={{\bf{F}}}_{{\rm{grad}}}+{{\bf{F}}}_{{\rm{rad}}}+{{\bf{F}}}_{{\rm{curl}}}\end{array}$$

where ${{\bf{p}}}_{e}^{O}$ is the electric orbital momentum, *n*_*m*_ is the refractive index of the medium, **P** is the Poynting vector, **S**_*e*_ is the electric part of the spin angular momentum, and *σ*_ext_ is the extinction cross-section. The first term on the right side of Eq. (3) is associated with the intensity gradient ($\nabla {|{\bf{E}}|}^{2}$) and is recognized as the conventional OGF (Fig. 3); The second term that correlates with **P** is the ORP; The last term lined to $\nabla \times {{\bf{S}}}_{e}$ is the spin-curl force.

**Fig. 4:**
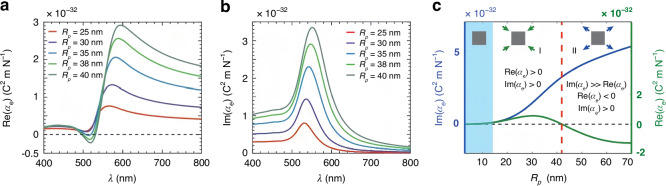
Polarizabilities of gold nanoparticles. **a** Real and (**b**) imaginary parts of the permittivity versus the wavelength for gold nanoparticles with various sizes. The real part of the permittivity can reverse its sign with the wavelength. **c** Real and imaginary parts of the permittivity versus the size of gold nanoparticle. The reversible Re(*α*_*e*_) indicates that the trapping and repelling OGF can be utilized for optical sorting. **a**–**c** Reproduced with permission^117^. Copyright 2020, WILEY-VCH Verlag GmbH & Co. KGaA, Weinheim

From Eq. (3), we observe that the optical force can be strongly influenced by the polarizabilities of the particle. The sign of Re(*α*_*e*_) is normally unaffected by the size or wavelength when the particle is dielectric, as it only possesses the real part of the refractive index or permittivity. In contrast, metallic particles, such as gold and silver nanoparticles, exhibit a profound wavelength-dependent imaginary part of the permittivity, which could lead to intriguing effects on Re(*α*_*e*_) and consequently the optical forces. Particularly, under certain conditions, the particle size and wavelength of light can reverse Re(*α*_*e*_), resulting in a repelling OGF on gold nanoparticles^117^, as shown in Fig. 4. However, Im(*α*_*e*_) can increase substantially when the particle is under the strong SPR, giving rise to a significantly enhanced ORP.

It is noted that, in the Rayleigh limit (*ka* $\ll$ 1), ${\alpha }_{e}^{0}$ and ${\alpha }_{m}^{0}$ may directly be employed instead of *α*_*e*_ and *α*_*m*_ for simplicity. Consequently, the OGF and ORP are given by

4 $${{\bf{F}}}_{{\rm{grad}}}=\frac{{\mathrm{Re}}({\alpha }_{e})}{4}\nabla {|{\bf{E}}|}^{2}=\frac{2\pi {n}_{2}{a}^{3}}{c}{\mathrm{Re}}\left(\frac{{m}^{2}-1}{{m}^{2}+2}\right)\nabla {I}_{l}$$

5 $$\begin{array}{l}{{\bf{F}}}_{{\rm{rad}}}=\frac{{\sigma }_{{\rm{ext}}}{n}_{m}}{c}{\bf{P}}={{\bf{F}}}_{{\rm{sca}}}+{{\bf{F}}}_{{\rm{abs}}}\\ \qquad=\frac{128{\pi }^{5}{n}_{2}{a}^{6}{{\bf{I}}}_{l}}{3c{\lambda }^{4}}{\mathrm{Re}}{(\frac{{m}^{2}-1}{{m}^{2}+2})}^{2}+\frac{8{\pi }^{2}{n}_{2}{a}^{3}{{\bf{I}}}_{l}}{c\lambda }{\text{Im}}(\frac{{m}^{2}-1}{{m}^{2}+2})\end{array}$$

where *c* is the speed of light in vacuum, **I**_*l*_ is the light intensity, $m={n}_{p}/{n}_{m}$, with *n*_*p*_ and *n*_*m*_ being refractive indices of the particle and medium, respectively. The ORP can be categorized into the scattering force and the absorption force (in the presence of particle absorption). Both the OGF and ORP are tightly correlated with the particle size and refractive index. Consequently, they can be utilized to differentiate and sort particles based on variations in these two properties. It is worth noting that Eq. (4) ignores the size effect by omitting the term of ${\alpha }_{e}^{0}$ in the denominator of Eq. (3). As a result, it may not accurately predict the sign reversal effect of the OGF with respect to size when the particle is metallic (see Fig. 4). However, it is convenient for predicting optical forces on dielectric nanoparticles or metallic nanoparticles that do not exhibit strong SPR.

Notably, when the electric field exhibits a spatially varying phase alongside a slowly varying amplitude, it can be expressed as

6 $${\bf{E}}({\bf{r}},t)={E}_{0}({\bf{r}}){{\bf{u}}}_{{\rm{pol}}}{e}^{i\varphi (r)}$$

where ${E}_{0}({\bf{r}})$ is the amplitude, ${{\bf{u}}}_{{\rm{pol}}}$ is the polarization vector, and *φ*(*r*) is the fast-varying phase. Substituting Eq. (6) into Eq. (3), the phase-gradient force **F**_phase_ is given as

7 $${{\bf{F}}}_{{\rm{phase}}}=\frac{1}{2}{\text{Im}}({\alpha }_{e}){E}_{0}^{2}\nabla \varphi$$

Evidently, particles of varying sizes and refractive indices undergo distinct phase-gradient forces, forming the fundamental physics behind sorting strategies utilizing this force.

### Optical pulling force and optical lateral force

The OPF denotes a counterintuitive force that acts in the opposite direction of the wavevector (Fig. 3c)^66^. This force can persist even without a negative Poynting vector. This intriguing force was first proposed theoretically by Chen et al. in 2011^81^. They investigated the optical force on a polystyrene sphere illuminated by a Bessel beam, which has a special wavefront and is non-diffractive over an extended focal length. The underlying physics is the enlarged forward scattering from the interference of radiation multipoles. Similar findings were also put forth in the same year by Novitsky et al., who linked the concept of the “tractor beam” (similar to the “OPF”) to the nonparaxial nature of the Bessel beam and the principle of momentum conservation^118^. Comprehensive reviews of the OPF can be found in refs. ^73,119^.

The Fourier decomposition of a beam comprises plane-wave components with *k*-vectors arranged in a cone at an angle *θ*_0_. The momentum of the incident photons is *ħk*cos*θ*_0_. Upon scattering, the recoiled photon momentum can be expressed as $\hslash k\langle \cos \theta \rangle$, where$\langle \cos \theta \rangle \,{\le}1$, representing the weighted-average direction of the scattered radiation. Thus, the optical force can be expressed as

8 $$F={W}_{{\rm{sca}}}{c}^{-1}(\cos {\theta }_{0}-\langle \cos \theta \rangle )$$

where *W*_sca_ is the scattered rate of the photon energy. The condition of the OPF is cos*θ*_0_ <1 and $\cos {\theta }_{0}-\langle \cos \theta \rangle \, {<}0$. For a plane wave, it is impossible to generate an OPF because cos*θ*_0_ = 1, whereas for a Bessel beam, cos*θ*_0_ <1, allowing for the potential generation of an OPF. This is a fundamental comprehension of the OPF, while further details on the multipole expansion of optical forces to elucidate this force can be found in ref. ^81^.

The OLF refers to a typical type of optical force that is perpendicular to the wavevector and irrelevant to the intensity or phase gradient (Fig. 3d)^35^. It is the consequence of the transfer of light transverse momenta from light to particle, encompassing both the transverse linear and angular momenta^35^. The OLF can originate from the transverse spin, spin momentum, imaginary Poynting momentum, chirality-light interaction, meta-robots, spin-orbit interaction, as well as many other effects. For further details, one can consult the recently published comprehensive review of the OLF^35^. Here, we provide a brief summary of how the OLF can be tailored for optical sorting purposes.

Compared with the conventional ORP and OGF, the OLF does not offer distinct capabilities in sorting particles with different sizes and refractive indices. However, it serves as a paradigm for sorting chiral particles^120^. For a chiral particle, its electric dipole moment and magnetic dipole moments satisfy

9 $$\left[\begin{array}{c}{\bf{p}}\\ {\bf{m}}\end{array}\right]=\left[\begin{array}{cc}{\alpha }_{ee} & i{\alpha }_{em}\\ -i{\alpha }_{em} & {\alpha }_{mm}\end{array}\right]\left[\begin{array}{c}{\bf{E}}\\ {\bf{H}}\end{array}\right]$$

where *α*_*ee*_, *α*_*mm*_ and *α*_*em*_ are polarizabilities for chiral dipolar particles, which are all correlated with the chirality parameter *κ*. When *κ* = 0, *α*_*ee*_ and *α*_*mm*_ are equal to the previously mentioned *α*_*e*_ and *α*_*m*_ for an achiral particle. Substituting Eq. (9) into Eq. (1), the optical force on a chiral particle is^16,121^

10 $$\begin{array}{ll}{{\bf{F}}}_{{\rm{chiral}}}=-\nabla U+\sigma {\bf{P}}+{\sigma }_{e}\nabla \times {{\bf{S}}}_{e}+{\sigma }_{m}\nabla \times {{\bf{S}}}_{m}+{\gamma }_{e}{\omega }^{2}{{\bf{S}}}_{e}+{\gamma }_{m}{\omega }^{2}{{\bf{S}}}_{m}\\ \qquad\qquad\,-{\text{Im}}({\alpha }_{em})\nabla \times {\bf{P}}+\frac{\omega {k}^{3}}{12\pi }[\text{Im}({\alpha }_{ee}{\alpha }_{mm}^{\ast })-\text{Im}({\alpha }_{em}{\alpha }_{em}^{\ast })]\text{Im}({\bf{E}}\times {{\bf{H}}}^{\ast })\end{array}$$

where

$$\begin{array}{ll}U=-\frac{1}{4}{\mathrm{Re}}({\alpha }_{ee}){|{\bf{E}}|}^{2}-\frac{1}{4}{\mathrm{Re}}({\alpha }_{mm}){|{\bf{H}}|}^{2}\\\qquad\,\,-\frac{1}{2}{\mathrm{Re}}({\alpha }_{em}){\text{Im}}({\bf{E}}\cdot {{\bf{H}}}^{\ast })\end{array}$$

$${{\bf{S}}}_{e}=\frac{{\varepsilon }_{0}}{4\pi \mu i}({\bf{E}}\times {{\bf{E}}}^{\ast }),\,{{\bf{S}}}_{m}=\frac{{\mu }_{0}}{4\pi \varepsilon i}({\bf{H}}\times {{\bf{H}}}^{\ast })$$

$${\sigma }_{e}=\omega \mu {\mu }_{0}{c}^{2}{\text{Im}}({\alpha }_{ee}),\,{\sigma }_{m}=\omega \varepsilon {\varepsilon }_{0}{c}^{2}{\text{Im}}({\alpha }_{mm})$$

$$\sigma =\frac{{\sigma }_{e}}{{c}^{2}}+\frac{{\sigma }_{m}}{{c}^{2}}-\frac{\omega {k}^{3}}{6\pi }[{\mathrm{Re}}({\alpha }_{ee}{\alpha }_{mm}^{\ast })+{\mathrm{Re}}({\alpha }_{em}{\alpha }_{em}^{\ast })]$$

$${\gamma }_{e}=-2\varepsilon \mu {\text{Im}}({\alpha }_{em})+\frac{{k}^{3}\mu }{3\pi {\varepsilon }_{0}}{\mathrm{Re}}({\alpha }_{ee}{\alpha }_{em}^{\ast })$$

$${\gamma }_{m}=-2\varepsilon \mu {\text{Im}}({\alpha }_{em})+\frac{{k}^{3}\varepsilon }{3\pi {\mu }_{0}}{\mathrm{Re}}({\alpha }_{em}{\alpha }_{mm}^{\ast })$$

Here, *U* is the potential energy; **S**_*e*_ and **S**_*m*_ represent spin angular momentum densities of electric and magnetic contributions, respectively; *σ* is the extinction cross-section. The first term on the right side of Eq. (10) is the OGF on a chiral particle, which is written as^16,121^

11 $${{\bf{F}}}_{{\rm{grad}}}=\frac{1}{4}\nabla [{\mathrm{Re}}({\alpha }_{ee}){|{\bf{E}}|}^{2}+{\mathrm{Re}}({\alpha }_{mm}){|{\bf{H}}|}^{2}+2{\mathrm{Re}}({\alpha }_{em}){\text{Im}}({\bf{E}}\cdot {{\bf{H}}}^{\ast })]$$

As we can see from Eq. (11), the OGF depends on the chirality parameter *κ*. Therefore, it can be used to distinguish chiral particles with different handedness^122^. For instance, in 2011, Cipparrone et al. discovered that left-handed and right-handed chiral particles exhibit distinct behaviours in response to polarized light, allowing for selective trapping or repulsion^123^. This approach was expanded upon by Tkachenko and Brasselet, who showcased the three-dimensional selective trapping and repulsion of liquid crystal chiral structures using circularly polarized Gaussian or Laguerre-Gaussian beams^124^. Recently, Yamanishi et al. unveiled that chiral OGFs can be influenced by the resonance and morphology of the particle^125^. Chiral OGFs can be enhanced in slotted waveguides, creating potential wells at distinct positions for left-handed and right-handed chiral particles^126^. The two types of chiral particles can move towards and be trapped in different potential wells, leading to the separation. Albeit some potential in separating chiral particles, trapping by OGFs only happens in a short range, e.g., a few wavelengths, causing inefficient sorting due to potential Brownian motion and difficulty in further isolation.

In addition to the OGF, sorting based on the ORP involves pushing chiral particles of different handedness by leveraging unique ORPs acting on them, which can be expressed as

12 $$\begin{array}{l}{{\bf{F}}}_{{\rm{rad}}}=\sigma {\bf{P}}=\left\{\!\right.\omega \mu {\mu }_{0}{\text{Im}}({\alpha }_{ee})+\omega \varepsilon {\varepsilon }_{0}{\text{Im}}({\alpha }_{mm})\\\qquad\qquad\quad-\,\frac{\omega {k}^{3}}{6\pi }[{\mathrm{Re}}({\alpha }_{ee}{\alpha }_{mm}^{\ast })+{\mathrm{Re}}({\alpha }_{em}{\alpha }_{em}^{\ast })]\left.\!\right\}{\bf{P}}\end{array}$$

In essence, the ORP also depends strongly on the chirality parameter *κ*, which is akin to the OGF. In 2013, Shang et al. calculated the ORP and torque on a chiral sphere in a Gaussian beam using generalized Lorent-Mie theory^127^. It was manifested that chiral particles with different handedness experience drastically distinct ORPs, paving the way for chiral sorting based on this force mechanism. Later, Tkachenko and Brasselet built up an experimental platform with two counter-propagating light beams to selectively push away chiral particles with different handedness by controlling light polarizations^128^. General formulations for optical forces on a spherical chiral particle in monochromatic optical fields were later presented by Zheng et al., which can be helpful in exploring chirality-dependent ORP and OGF^122^. Similar to the conventional ORP, the chiral ORP is also mainly used to sort microchiral particles. It is of great importance to develop methods that can handle nanoscaled chiral particles with nanometer precisions.

We then get back to Eq. (10), besides the first and second terms representing the OGF and ORP, respectively, the remaining terms have the potential to induce fascinating OLF. In fact, the OLF can also be induced from the lateral linear momentum **P** by the effect of an interface that breaks the mirror symmetry of the system^69,72^. According to Eq. (10), the OLF can be induced from the transverse spin momentum (called the Belinfante spin momentum) by $\nabla \times {\bf{S}}$, transvers spin **S**, energy vortex by $\nabla \times {\bf{P}}$, as well as the imaginary Poynting momentum $\text{Im}({\bf{E}}\times {{\bf{H}}}^{\ast })$. The OLF is influenced by various factors, that could significantly improve the sorting size, resolution and speed.

Let us revisit the polarizabilities ${\alpha }_{ee}$, ${\alpha }_{mm}$ and ${\alpha }_{em}$ for chiral particles in Eq. (9), which can be expressed according to Mie coefficients ${a}_{1}$, ${b}_{1}$, ${c}_{1}$ as^34,75,129^

13 $$\begin{array}{ll}{\alpha }_{ee}=\frac{i6\pi \varepsilon {\varepsilon }_{0}}{{k}^{3}}{a}_{1},\,{\alpha }_{mm}=\frac{i6\pi \mu {\mu }_{0}}{{k}^{3}}{b}_{1}\\{\alpha }_{em}=\frac{6\pi }{\omega {k}^{2}}{c}_{1}=-\frac{6\pi \sqrt{\varepsilon \mu }}{c{k}^{3}}{d}_{1}\end{array}$$

The scattering coefficients can be defined as^16,130^

14 $${a}_{n}=\frac{{V}_{n}(R){A}_{n}(L)+{V}_{n}(L){A}_{n}(R)}{{W}_{n}(L){V}_{n}(R)+{W}_{n}(R){V}_{n}(L)}$$

15 $${b}_{n}=\frac{{W}_{n}(L){B}_{n}(R)+{W}_{n}(R){B}_{n}(L)}{{W}_{n}(L){V}_{n}(R)+{W}_{n}(R){V}_{n}(L)}$$

16 $${c}_{n}=-{d}_{n}=i\frac{{W}_{n}(R){A}_{n}(L)-{W}_{n}(L){A}_{n}(R)}{{W}_{n}(L){V}_{n}(R)+{W}_{n}(R){V}_{n}(L)}$$

with

17 $${W}_{n}(J)={m}_{a}{\psi }_{n}({m}_{J}X){\xi}_{n}^{\prime}(X)-{\xi}_{n}(X){\psi}_{n}^{\prime}({m}_{J}X)$$

18 $${V}_{n}(J)={\psi}_{n}({m}_{J}X){\xi}_{n}^{\prime}(X)-{m}_{a}{\xi}_{n}(X){\psi}_{n}^{\prime}({m}_{J}X)$$

19 $${A}_{n}(J)={m}_{a}{\psi }_{n}({m}_{J}X){\psi }_{n}^{{\prime} }(X)-{\psi }_{n}(X){\psi }_{n}^{{\prime} }({m}_{J}X)$$

20 $${B}_{n}(J)={\psi }_{n}({m}_{J}X){\psi }_{n}^{{\prime} }(X)-{m}_{a}{\psi }_{n}(X){\psi }_{n}^{{\prime} }({m}_{J}X)$$

Here, $J=L,R$ stands for the left-handed and right-handed chirality, respectively, and $X=kr$. ${\psi }_{n}(\rho )=\rho {j}_{n}(\rho )$, ${\xi }_{n}(\rho )=\rho {h}_{n}^{(1)}(\rho )$, where ${j}_{n}(\rho )$ is the spherical Bessel function, and ${h}_{n}^{(1)}(\rho )$ is the first type of spherical Hankel function. ${\varepsilon }_{p}$ and ${\mu }_{p}$ are the relative permittivity and permeability of the particle, respectively. And the relative refractive indices ${m}_{L}$, ${m}_{R}$ and the average refractive index ${m}_{a}$ can be expressed as ${m}_{L}=\sqrt{{\varepsilon }_{p}{\mu }_{p}/\varepsilon \mu }+\kappa$, ${m}_{R}=\sqrt{{\varepsilon }_{p}{\mu }_{p}/\varepsilon \mu }-\kappa$, and ${m}_{a}=({m}_{L}+{m}_{R})/2$.

To simulate particle dynamics in intricate light fields such as topological optical fields and interference waves, one may refer to the well-known Langevin equation, which is given as^7,131–133^

21 $$m\frac{d{\bf{v}}}{dt}+6\pi \eta a({\bf{v}}-{\bf{u}})+{{\bf{F}}}_{{\rm{opt}}}+{{\bf{F}}}_{{\rm{Brownian}}}=0$$

where **u** is the flow velocity, *m*, *a* and **v** are the mass, radius and velocity of the particle, respectively; *η* is the viscosity of the medium; **F**_opt_ and **F**_Brownian_ are optical force and Brownian force, respectively. The first term of Eq. (21) denotes the inertial term of the nanoparticle; the second term is the fluidic drag force^134–136^. **F**_Brownian_ is the random force with zero mean, which can be written as ${{\bf{F}}}_{{\rm{Brownian}}}=\sqrt{2S}{\bf{W}}(t)$, where **W**(*t*) is a white noise with $\langle {\bf{W}}(t)\rangle =0$, and 2*S* is the intensity of the noise.

The simulation of the trajectory of the particle can be conducted in the commercial software Matlab or COMSOL by solving Eq. (21). In this scenario, the matrix of optical force is required, which can be rigorously addressed using the Maxwell stress tensor in the Minkowski’s form as^85,137^22

23 $$\langle {T}_{ij}\rangle =\frac{1}{2}[{D}_{i}{E}_{j}^{\ast }+{B}_{i}{H}_{j}^{\ast }-\frac{1}{2}({\bf{D}}\cdot {{\bf{E}}}^{\ast }+{\bf{B}}\cdot {{\bf{H}}}^{\ast }){\delta }_{ij}]$$

where $\hat{n}$ is the unit outward normal to the integral surface, *A* is the boundary enclosing the particle for the integral, and ${\delta }_{ij}$ is Kronecker delta; **D** = *ε***E** and **B** = *µ***H**, where *ε* and *µ* are the electric permittivity and magnetic permeability of the medium, respectively. It is worth noting that the accuracy of force calculations is closely tied to mesh/step refinement, particularly when there are significant variations in the light field in the near field. Therefore, striking a balance between mesh complexity and computational workload is crucial at the outset of the calculation process.

In the following two sections, we will delve into various sorting strategies in detail from the viewpoints of active and passive sorting. We will elucidate the characteristics of different methods and discuss their limitations, effectiveness, speeds, and other pertinent factors.

## Active optical sorting

### Sorting using digital image processing

The image processing techniques applied to cell sorting can be roughly divided into two sub-categories: traditional image processing techniques and machine learning-based image processing techniques^138–140^. Traditional image processing is based on predefined rules and algorithms, utilizing handcrafted features to solve well-defined tasks such as thresholding^141–143^, edge detection^144–146^, and morphological operations^147,148^. These methods lack the adaptability to changing conditions and often require manual intervention for new scenarios. In contrast, machine learning-based image processing relies on data-driven approaches, learning patterns directly from data during training. Typical models include Convolutional Neural Networks (CNNs)^149–152^ or SVMs^153–156^ that automatically extract relevant features, thus demonstrating adaptability to variations in data. Unlike traditional techniques that are transparent and explainable, machine learning models are regarded as black-box systems, in which explaining the decision-making processes is challenging. The flexibility and adaptability of machine learning come at the cost of relying on massive labelled datasets for training. In practice, collaborative approaches combine task-specific traditional image processing with the data-driven power of machine learning to achieve comprehensive and robust image analysis.

**Fig. 5:**
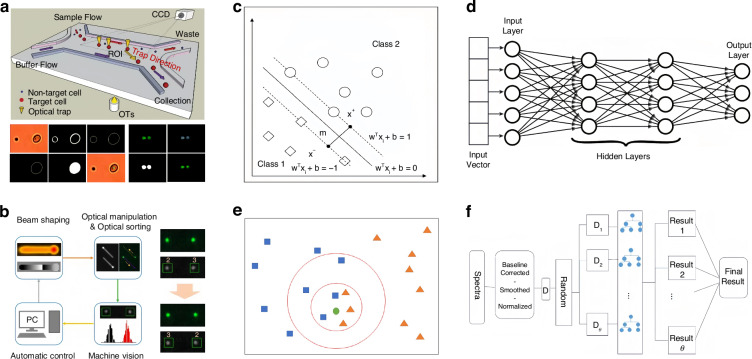
Traditional image processing and machine learning-based image processing techniques in cell sorting. **a** Traditional threshold segmentation approach for edge detection and identification of target cells. Reproduced with permission^105^. Copyright 2011, The Royal Society of Chemistry. **b** An intensity-phase-based beam-shaping algorithm can be utilized to identify individual metal nanoparticles based on pixel intensity, enabling effective sorting. Reproduced with permission^65^. Copyright 2020, American Chemical Society. **c** Support Vector Machines (SVM) are utilized for the classification of human chromosomes^108^. Reproduced with permission^108^. Copyright 2017, Elsevier Ltd. **d** Multilayer Perceptrons (MLP) can effectively differentiate between healthy and diseased cells by analysing their mechanical properties. Reproduced with permission^109^. Copyright 2023, Springer Nature. **e** K-Nearest Neighbor (KNN) can be used to identify epileptic components. Reproduced with permission^110^. Copyright 2022, Wiley Periodicals LLC. **f** Random Forest can accurately classify between breast cancer and normal single cells. Reproduced with permission^112^. Copyright 2020, Elsevier B.V

To be specific, traditional image processing techniques function in cell sorting across multiple phases, addressing tasks like edge detection^157^, segmentation^158^, feature extraction^159^, and quality control^160^. In this context, diverse methods have been employed. Thresholding^161–164^ is a fundamental technique that sets a threshold value to differentiate between foreground (cells) and background for each pixel in an image based on its intensity. This technique is commonly utilized for fundamental segmentation in cell sorting^165–168^. Mathematically, thresholding can be represented as

24 $$B(x,y)=\left\{\begin{array}{cc}1, & {\rm{if}}\,I(x,y) > T\\ 0, & {\rm{otherwise}}\end{array}\right.$$

where $B(x,y)$ represents the binary image, $I(x,y)$ represents the intensity of the pixel at coordinates $(x,y)$, and $T$ represents the threshold value. Wang et al. employed a traditional threshold segmentation approach to identify target cells, subsequently using a laser trap to transport them to their designated locations for sorting yeast cells and human embryonic stem cells^105^. The authors converted original colour images of microbeads (with a diameter of 2 µm) and yeast cells (with a diameter of 5 – 8 µm) into grey images. Particles were distinguished from the background using a threshold segmentation algorithm. To differentiate target particles from those of similar size, fluorescence images are binarized by setting a threshold on the fluorescence intensity, as illustrated in Fig. 5a. Edge detection^169^, a technique that employs algorithms like the Sobel operator^170–172^ or Canny edge detector^173–175^, is employed to identify boundaries between objects. The Sobel operator, for example, computes the gradient magnitude of an image to detect edges, which are regions of significant intensity variation. The operator consists of two convolution kernels, one for computing the gradient approximation in the horizontal direction (**G**_*x*_) and the other in the vertical direction (**G**_*y*_)

25 $${{\bf{G}}}_{x}=\left[\begin{array}{ccc}1 & 0 & -1\\ 2 & 0 & -2\\ 1 & 0 & -1\end{array}\right]\ast {\bf{I}},{{\bf{G}}}_{y}=\left[\begin{array}{ccc}1 & 2 & 1\\ 0 & 0 & 0\\ -1 & -2 & -1\end{array}\right]\ast {\bf{I}}$$

Here, * denotes the convolution operation, and **I** represents the input image. These kernels are typically applied to the image to compute the gradient approximations in both directions simultaneously. After applying the Sobel operator in both directions, the gradient magnitude is computed as the square root of the sum of the squared gradients

26 $${\bf{G}}=\sqrt{{{\bf{G}}}_{x}^{2}+{{\bf{G}}}_{y}^{2}}$$

This results in an image where edges are highlighted as peaks in the gradient magnitude. The direction of the gradient at each point can also be computed using the arctangent function $\arctan ({{\bf{G}}}_{y}/{{\bf{G}}}_{x})$. The Sobel operator provides a simple yet effective method for detecting edges in images, thus helping define cell boundaries and identify crucial features related to cell shape in cell sorting^176^. Nan and Yan moulded the beam based on both intensity and phase, employing machine vision for the real-time adjustment of potential wells^65^. This ensures that particles remain in the light field through a combination of phase gradient and intensity gradient forces. The illuminating light is processed into a circular beam using dark-field imaging optics, facilitating precise observation of the particle edges in the field of view, similar to edge detection in image processing. By analysing the scattering intensity, converting the dark-field image to grey, and examining pixel intensity, it is possible to identify the size and material composition of metal particles. Notably, they effectively sorted gold and silver particles with a diameter of 150 nm, as shown in Fig. 5b. Other techniques such as the morphological operations^147,148^, including erosion, dilation, opening, and closing, are employed to modify the shape and structure of objects in images, refine cell boundaries, remove noise, and separate clustered cells in cell sorting. Furthermore, Blob analysis^177^ is a valuable technique in cell sorting for detecting and analysing individual cells, particularly when cells are well-separated. It involves identifying and analysing connected regions of interest (blobs) in an image. The Hough transform^174,178^ can be used to identify circular objects such as cells, while the histogram analysis^179,180^ can enhance contrast and adjust brightness to improve the visibility of cell features.

While traditional image processing techniques remain important, their integration with machine learning approaches enhances the accuracy and robustness of cell sorting, thus advancing biological and medical research applications. Machine learning techniques play a critical role in automating and enhancing cell sorting processes, enabling efficient classification of cells based on various features. In cell classification, supervised learning methods such as SVMs^108,181–184^ and neural networks^185–188^ (like MLPs) are trained on labelled datasets to categorize cells based on the type, health status, or other relevant characteristics. The MLP is a fundamental algorithm widely used for classification and regression. The algorithm involves several key steps. The weights and bias of the network are first initialized randomly. Then, the weighted sum of inputs to each node in each layer is calculated during forward propagation, and the output of each node is generated after passing through a point-wise activation function. This can be mathematically represented as

27a $${w}_{jk}^{l}=\mathop{\sum }\limits_{k=1}^{{n}^{l-1}}{w}_{jk}^{l}{a}_{k}^{l-1}+{b}_{j}^{l}$$

27b $${a}_{j}^{l}=\sigma ({z}_{j}^{l})$$

where ${z}_{j}^{l}$ is the weighted sum of inputs for node $j$ in layer $l$, ${w}_{jk}^{l}$ represents the weight of the connection between node $k$ in layer $l-1$ and node $j$ in layer $l$, ${a}_{k}^{l-1}$ is the output of node $k$ in the previous layer, ${b}_{j}^{l}$ is the bias for node $j$ in layer $l$, $\sigma$ is the activation function, and ${a}_{j}^{l}$ is the output of node $j$ in layer $l$. This process is repeated in the hidden layers until reaching the final output layer, producing the predictions of the network. Subsequently, the error between the predicted output and the actual target output is calculated using the selected loss function. Backpropagation then computes the gradient of the error to the network weights and biases, allowing tuning using optimization algorithms such as gradient descent. This iterative process continues until the network converges to a solution that minimizes the error, thereby training the MLP to make accurate predictions. Davidovic et al. employed an MLP to differentiate between damaged and intact Saccharomyces cerevisiae cells, achieving the highest classification accuracy among the methods tested^185^. The MLP can differentiate between healthy and diseased cells based on their mechanical properties^109^, as shown in Fig. 5d. Li et al. employed the MLP to predict blood cell types^186^. by establishing a correlation between a set of input features and a specified dependent variable. SVM is another powerful supervised learning model, that aims to find the optimal hyperplane that separates the data point into different classes in the feature space. Given a set of training examples $({{\boldsymbol{x}}}_{i},{y}_{i})$, where ${{\boldsymbol{x}}}_{i}$ represents the feature vector and ${y}_{i}$ is the class label ${y}_{i}\in \{-1,+1\}$, SVM seeks to maximize the margin between the hyperplane and the nearest data points, known as support vectors. The decision function of an SVM is defined as

28 $$f(x)=sign\left(\mathop{\sum }\limits_{i=1}^{N}{\alpha }_{i}{y}_{i}{{\boldsymbol{x}}}_{i}^{T}{\boldsymbol{x}}+b\right)$$

where $N$ is the number of support vectors, ${\alpha }_{i}$ are the Lagrange multipliers obtained by solving the optimization problem, ${y}_{i}$ are the class labels, ${{\boldsymbol{x}}}_{i}$ are the support vectors, and $b$ is the bias term. The optimization objective involves minimizing $\frac{1}{2}{\Vert {\boldsymbol{w}}\Vert }^{2}$, subject to the constraint ${y}_{i}({\boldsymbol{w}}\cdot {{\boldsymbol{x}}}_{i}+b)\ge 1$, where ${\boldsymbol{w}}$ is the weight vector perpendicular to the hyperplane. SVM can handle nonlinearly separable data by transforming the input space into a higher-dimensional feature space using polynomial or radial basis function kernels, which allows for the separation of classes by nonlinear decision boundaries. Researchers have effectively employed the SVM to differentiate between patients with and without decompensation^181^, identify breast cancer tumours^182^, and classify tumours as malignant, critical, or benign^183^. Kusakci et al. curated and trained datasets, utilizing the numerical optimization to determine optimal parameters of the SVM for classifying 23 pairs of human chromosomes, thereby assisting in the diagnosis of genetic disorders^108^, as shown in Fig. 5c.

Deep learning^189^, especially the CNN, has manifested effectiveness in the image-based cell classification^190–196^, with transfer learning being a valuable approach in scenarios with limited labelled data^197,198^. CNN is designed to learn spatial hierarchies of features automatically and adaptively from raw pixel data. The fundamental building block of a CNN is the convolution layer, which applies convolution operations to the input image using learnable filters or kernels. Mathematically, the output feature map *C* of a convolutional layer is computed as

29 $${C}_{i,j}=\sigma \left({\sum}_{m}{\sum}_{n}{I}_{i+m,i+n}\times {W}_{m,n}+b\right)$$

where $I$ represents the input image, $W$ denotes the learnable convolutional filter, $b$ is the bias term, and $\sigma$ is the activation function. Pooling layers, such as max pooling or average pooling, are often inserted between convolutional layers to downsample the feature maps, reducing spatial dimensions and the number of parameters. After several convolutional and pooling layers, the output is flattened and passed through one or more fully connected layers, where the output $O$ is calculated as

30 $$O=\sigma (W\cdot X+b)$$

where $W$ represents the weight matrix, $X$ is the input vector obtained by flattening the feature maps, $b$ is the bias and $\sigma$ is the activation function. CNNs are trained using backpropagation and optimization algorithms to minimize a loss function, enabling them to learn the hierarchy representation of visual data and achieve state-of-the-art performance in tasks like image classification, object detection, and image segmentation^199,200^. In cell sorting, Togacar et al. used a CNN to classify leukocyte subtypes^201^, including eosinophils, lymphocytes, monocytes, and neutrophils, achieving an accuracy of 97.78%. This methodology holds promise for disease assessment and diagnosis in patients. Recently, Jeon et al. utilized acoustic tweezers to capture and measure backscattered signals from various cell types and polystyrene microbeads^202^. They employed a CNN to denoise raw signals, extract features in time, frequency, and time-frequency domains, and classified micrometre-sized red blood cells and normal SV40 immortalized epithelial prostate cells. Furthermore, CNNs have been widely employed for the classification of leukocyte subtypes^203,204^ and the detection of lung cancer cells^205^, among other applications. Go et al. utilized a non-parametric and supervised algorithm, the KNN, to build a classification model^206^. The model successfully identified the various types of erythrocytes present in holographic images, including intervertebral disc cells, spine-forming cells, and spherical erythrocytes, with an accuracy rate larger than 97%. Moreover, KNN manifests its versatility in predicting five types of membrane proteins^207^, distinguishing between cancerous and non-cancerous samples^208^, classifying cells with different phenotypes^209^, differentiating between three subtypes of malignant lymphoma^210^, and classifying Pseudomonas aeruginosa^211^. As illustrated in Fig. 5e, the KNN algorithm showcases its capability in identifying the epileptic component^110^.

In addition to supervised learning, unsupervised learning techniques such as autoencoders can facilitate accurate feature extraction for capturing essential features in cell classification. Dimensionality reduction methods like Principal Component Analysis and t-Distributed Stochastic Neighbor Embedding can help visualize the relationships between cells. Real-time decision-making benefits from online learning and reinforcement learning, allowing models to adapt dynamically to changing conditions during cell sorting. Methods like Random Forests and cost-sensitive learning techniques prioritize accurate classification of critical cell types, addressing common imbalanced datasets in cell sorting^212–221^. Illustrated in Fig. 5f, Shen et al. utilized the random forest algorithm to distinguish single cells as either breast cancer or normal^112^. Asthma, a chronic airway disease, can be diagnosed by analysing miRNAs from eosinophils, which act as markers. Rodrigo-Muñoz et al. developed a random forest model to classify asthma severity using a set of miRNA variables in an unsupervised manner^222^. As interpretability becomes crucial, explainable AI methods like LIME (Local Interpretable Model-agnostic Explanations)^223^ or SHAP (SHapley Additive exPlanations)^224^ can be employed to elucidate decisions made by complex machine learning models in cell sorting. Machine learning techniques, spanning traditional algorithms to advanced deep learning, play a significant role in enhancing the efficiency and accuracy of cell sorting processes. This establishes machine learning as a fundamental component in modern biological and medical research^225,226^.

### Fluorescent labelling-assisted sorting

With the assistance of image processing, target particles are labelled with fluorescence staining and subsequently sorted using optical tweezers. Fluorescent staining techniques can be categorized into cell surface staining and intracellular staining. Cell surface staining encompasses cell membrane staining^93,94^ and immunofluorescence techniques^99^, allowing researchers to selectively label specific components or structures on the surface of cells or particles. By leveraging these fluorescence labelling methods, precise identification and tracking of target particles for subsequent manipulation and sorting can be achieved using optical tweezers. The integrated approach of combining fluorescence staining and optical tweezers provides a powerful tool for precise particle manipulation and sorting in a range of research and application scenarios.

Wheat germ agglutinin^94,227–230^ can be used to label cell membranes of different types of cells due to its property of coupling to N-acetyl-β-D-glucosaminyl residues and N-acetyl-β-D-glucosaminyl oligomers. This reagent is commonly used to label cell membranes of mammalian cells^231^, Gram-positive bacteria^232^, and yeasts^233^, as well as skeletal^234^ and cardiac sacral membranes^235^. CellMask Membrane Dye^93,228,236^, with low toxicity and no impact on mammalian cell function, can be used to label red fluorescent proteins onto cell membranes.

**Fig. 6:**
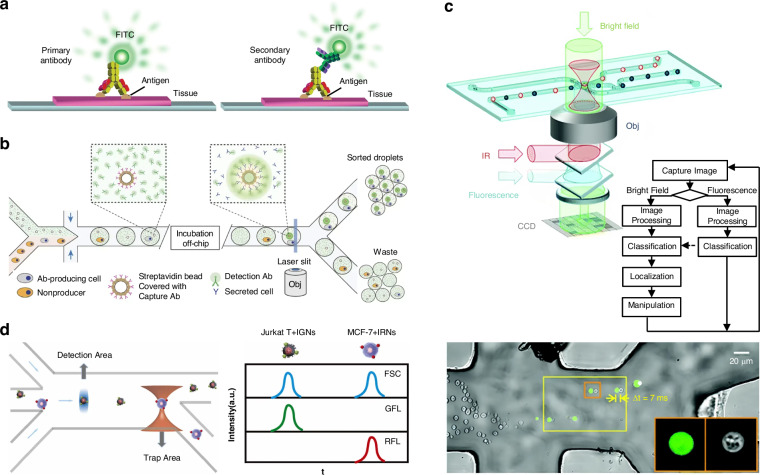
Fluorescent labelling-assisted sorting. **a** Fluorescent staining of antibodies is accomplished through both direct and indirect methods. Reproduced with permission^240^. Copyright 2022, Springer Nature. **b** The fluorescent labelling sorting process. Reproduced with permission^241^. Copyright 2013, Springer Nature. **c** Fluorescent identification and sorting of mitochondria, yeast cells, and rod-shaped bacteria. Reproduced with permission^266^. Copyright 2012, Royal Society of Chemistry. **d** Specific labelling of two tumour cells was achieved through the utilization of fluorescent quantum dots incorporated within two-colour fluorescent nanospheres, enabling effective cell sorting by resolving fluorescence signals. Reproduced with permission^101^. Copyright 2022, Elsevier B.V

Immunofluorescence relies on the specific binding of antigens and antibodies, leading to the fluorescent labelling^237–239^. The labelled substances bind specifically to the corresponding antigen or antibody, and the fluorescence is observed under a fluorescence microscope. Fluorescence microscopy is a technique that involves labelling antibodies or antigens with fluorescent substances. Currently, labelling antibodies with fluorescent substances is a common practice. Figure 6a illustrates two methods for detecting antigens: direct and indirect^240^. The direct method involves labelling the antibody corresponding to the antigen with a fluorescent substance and observing the fluorescence upon reacting with the antigen. The indirect method involves reacting the unlabelled primary antibody with the antigen and then observing the fluorescence using the anti-globulin antibody labelled with the fluorescent substance (the secondary antibody) which binds specifically to the primary antibody. Steps involved in this process include sample preparation, fixation, permeabilization, closure, primary antibody incubation, secondary antibody incubation, re-staining of the nucleus, and sealing of the film for fluorescence observation. Figure 6b illustrates the sorting process with the fluorescent labelling using the droplet microfluidics^241^. Compartmentalization of single cells in droplets facilitates the analysis of proteins released from or secreted by cells, which is unattainable by conventional flow cytometry. A binding assay for detecting antibodies secreted from single mouse hybridoma cells was proposed to detect secreted antibodies using single beads coated with anti-mouse IgG antibodies in droplets. The bead can capture antibodies and subsequently emit fluorescence signals for observation. The fluorescent sorting technique can be performed at approximately 200 Hz.

The staining of intracellular organelles such as lysosomes^242,243^, nucleolus^96,244^, mitochondrion^245,246^, Golgi complex^97,247^, and endoplasmic reticulum^248,249^ can be used to label target cells. LysoTracker and CellLight Lysosomal Fusion Protein are two examples of probes that can be used to label lysosomes^250–253^. LysoTracker is highly selective for acidic organelles^95^, while CellLight Lysosomal Fusion Protein can be used to label lysosomes in living cells and track their dynamics^254^. Live ReadyProbes reagents are used to sort cells based on their DNA content. CellLight nucleoprotein labelling reagents are almost non-toxic, non-chemically destructive, and safe for living cells. HCS NuclearMask dyes detect DNA content in both living and formaldehyde-fixed cells and can also delineate cell boundaries^228,254–256^. CellLight reagents are suitable for mitochondria in living cells^254,257,258^, whereas caution should be exercised when using MitoTracker probes as they may impact cell survival and trigger apoptosis^259–261^. CellLight protein labelling technology targets the Golgi complex using fluorescent fusion proteins^254,262^, and ceramide living cell dyes have low toxicity^254^. ER-Tracker endoplasmic reticulum dyes are highly selective and cell-permeable^263,264^. CellLight reagents are suitable for labelling the Endoplasmic Reticulum to track cellular dynamics. CellLight protein labelling technology can be used in conjunction with other fluorescent dyes for co-localization studies on living or post-fixed cells^254^.

Fluorescent labelling and optical tweezers have been employed to identify and sort target cells. Wang et al. gathered cells into a narrow streamline, distinguished the target cells by detecting their fluorescent properties, and then used optical tweezers to divert the target cells to the designated channel, while allowing the other cells to flow with the fluid to the waste channel^265^. The sorting of mammalian cells is achieved by applying laser energy to cells that is 2–3 orders of magnitude lower than their damage threshold, thus preserving their activity. Figure 6c demonstrates that cell types were differentiated by transmission intensities in the bright field, while mitochondria, yeast cells, and rod-shaped bacteria were sorted based on fluorescence intensities in the fluorescence channel^266^. Utilizing a diode laser bar, it was possible to locate particles in the line trap. The particles were stained with two different fluorescent dyes and their fluorescent properties were identified at the point of output release using a waveguide. This allowed for sorting particles ranging from 4 to 10 µm into the desired output stream^267^.

Quantum dots are a type of fluorescent nanomaterials, characterized by their semiconductor nature and typically ranging in diameter from 2 to 10 nm^268–271^. One of their defining features is their exceptional fluorescence properties. In acknowledgement of their groundbreaking work in the “discovery and synthesis of quantum dots”, Moungi G. Bawendi, Louis E. Brus, and Alexei I. Ekimov were honoured with the Nobel Prize in Chemistry in 2023. Varying in size, quantum dots exhibit distinct colours when exposed to laser irradiation, showcasing the benefits of single-element excitation for multiple emissions, high fluorescence intensity, and excellent stability. As shown in Fig. 6d, Zheng et al. designed a fluorescence-activated cell sorting platform. Due to notable variations in protein expression levels on the cell surfaces of two distinct cell types, a targeted immunolabeling approach was employed. Quantum dots were encapsulated within two-colour fluorescent nanospheres to specifically label the two cell types. Subsequently, the distinct two-colour fluorescence signals were resolved, enabling the effective sorting of tumour cells^101^.

### Raman spectra-assisted sorting

**Fig. 7:**
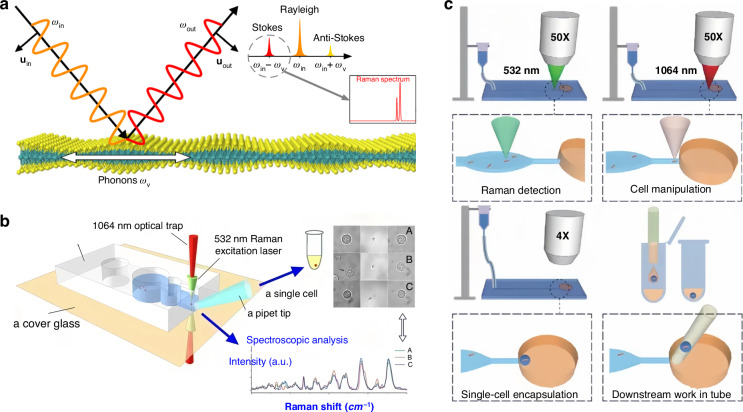
Raman spectra-assisted sorting. **a** Principle of the Raman scattering. Reproduced with permission^272^. Copyright 2020, Springer Nature. **b** Raman identification for the separation of single cells. Reproduced with permission^282^. Copyright 2019, American Chemical Society. **c** Utilizing Raman optical tweezers for the sorting of individual bacterial cells and conducting phenome-genome profiling on them. Reproduced with permission^284^. Copyright 2020, WILEY-VCH Verlag GmbH & Co. KGaA, Weinheim

The Raman effect, unveiled by Indian physicist Sir Chandrasekhara Venkata Raman in 1928^88^ and honoured with the Nobel Prize in 1930^88^, delineates a fascinating phenomenon where the energy of scattered photons diverges from that of incident photons^272^, as depicted in Fig. 7a. These distinct Raman signals serve as “molecular fingerprints”^273^, enabling the quantitative differentiation and characterization of various substances through their unique Raman spectra. Raman spectroscopy has demonstrated effectiveness in cancer diagnosis^274^, bioanalysis^275^, solar cell development^276^, and material characterization^277,278^. Notably, it can also be employed for real-time monitoring and control of chemical processes such as petrochemicals, pharmaceuticals, and polymers^279,280^.

In 1984, Thurn pioneered the integration of Raman spectroscopy with optical tweezers to confine micrometre-sized particles (10 – 30 µm) within an optical potential well, thus enabling the generation of Raman spectra^281^. This innovation, termed the Raman microprobe, marked a significant advancement in analytical techniques^281^. Subsequently, Fang et al. separated individual cells using Raman spectroscopy and OTs, as shown in Fig. 7b, illustrating the extraction of single cells, including BGC823 gastric cancer cells, erythrocytes, lymphocytes, and Escherichia coli, using a 1064 nm laser (serving as the light source for optical tweezers) and a 532 nm laser (Raman laser exciter) in a non-destructive identification. The success rate of isolation reached 90%^282^. In the same year, Parlatan et al. used holographic optical tweezers to generate multiple potential wells, facilitating the identification of multi-particle Raman signals and the sorting of micron-sized biological particles^283^.

The precise sorting of individual cells holds significant importance in analysing phenotypes and genotypes of single cells, thereby unravelling the intricate mechanisms underlying biological systems and processes. Recently, Xu et al. presented an interesting approach termed Raman-Activated Gravity-driven single-cell Encapsulation and Sequencing. This method integrated Raman optical tweezers with gravity and capillarity to facilitate the sorting, genome sequencing, and culturing of single bacterial cells^284^, as shown in Fig. 7c. Raman optical tweezers also have the capability to extract and characterize graphene flakes and carbon nanotubes. By combining optical trapping with spectroscopic analysis to probe the structure of individual graphene flakes and analyse their Brownian motion, Maragó et al. provided insights into the optical trapping of two-dimensional structures that paved the way for all-optical sorting of biological membranes and anisotropic macromolecules^285^. To determine the structure of single-layer graphene flakes, the target graphene flake was initially probed, followed by capturing the monolayer graphene flake using a laser power of 1 – 2 mW. Subsequently, the flake was pulled into the optical trap, and its Brownian motion was examined in the optical trap. Wu et al. employed standing-wave Raman tweezers with a low laser power to stabilize and characterize nanoparticles of various materials. The technique led to a 4 – 8 fold increase in the Raman signal, facilitating the sorting of specific single-walled carbon nanotubes^103^.

**Fig. 8:**
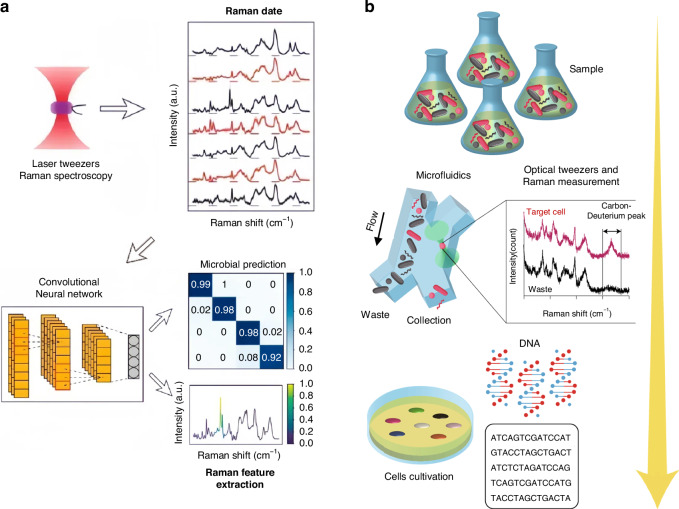
Combination of Raman tweezers and deep learning for classifying and sorting. **a** Combining Raman spectroscopy, optical tweezers, and ConvNet for microbial classification. Reproduced with permission^288^. Copyright 2020, American Chemical Society. **b** The integration of microfluidics, optical tweezers, and the K-means clustering algorithm that aims to differentiate Raman spectra enables the active sorting of cells and their further characterization. Reproduced with permission^289^. Copyright 2020, Springer Nature

The integration of deep learning algorithms has injected new vigour and vitality into the processing and differentiation of Raman spectra from various cells^286,287^. As shown in Fig. 8a, Lu et al. extracted the Raman spectra of microorganisms using Raman optical tweezers^288^. The trained ConvNet classification model can identify and classify different types of microbial with an extremely high accuracy. For example, the classification accuracy of the bacterium *E. coli* and the archaea *H. mediterranean* is as high as 100%. Lee et al. employed optical tweezers within a microfluidic device to manipulate individual cells for the purpose of measuring their Raman spectra. They utilized a machine-learning algorithm, specifically K-means clustering, to categorize a blend of cells. Subsequently, they conducted culturing and characterization of the sorted cells^289^, as depicted in Fig. 8b. In addition, the combination of laser tweezers Raman spectroscopy and deep learning can be utilized to identify liver cancer cells^290^ and classify marine bacteria^291^.

## Passive optical sorting

### Sorting by the optical radiation pressure

**Fig. 9:**
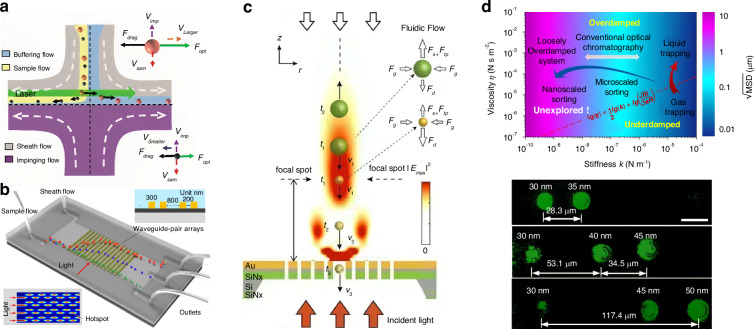
Sorting based on the optical radiation pressure. **a** Optical chromatography for sorting gold nanoparticles in a stagnant region in microfluidics. Reproduced with permission^297^. Copyright 2016, American Chemical Society. **b** Creation of a near-field optical lattice to sort polystyrene nanoparticles by leveraging the distinct lateral displacements of particles of varying sizes within the near-field hotspot array. Reproduced with permission^298^. Copyright 2021, Elsevier B.V. **c** Optical chromatography is employed to sort exosomes (less than 200 nm in diameter) by focusing a plane wave through a plasma nanopore array. Reproduced with permission^302^. Copyright 2019, Springer Nature. **d** The synergy of a quasi-Bessel beam and the flow creates a loosely overdamped system, enabling the trapping of nanoparticles at different locations for separation. Reproduced with permission^7^. Copyright 2018, AAAS

The ORP was discovered hundreds of years ago, when Kepler reported the hypothesis that light carries momentum which could induce an ORP on objects in 1619. This hypothesis was later proved by Maxwell’s equations in 1873. Intriguingly, momenta of electromagnetic fields in different media also give rise to the well-known Abraham-Minkowski controversy^34,292–296^. The ORP was first employed by Ashkin in the 1970s to propel particles and was used to confine particles using two counter-propagating light beams^44^. As shown in the roadmap of Fig. 2, the pioneer work^54^ using the ORP to sort microparticles was performed by Buican et al. in 1987. With the assistance of optofluidic chip design and hydrodynamics, Wu et al. created a stagnant region where 100 nm gold nanoparticles can be deflected from their previous path by the SPR-enhanced ORP, thus enabling their separation from 50 nm ones^297^, as shown in Fig. 9a. This method provides a rapid and stable platform for sorting metallic nanoparticles, showing potential in clinical diagnostics. A similar strategy to deflect various dielectric nanoparticles to their respective outlets can also be implemented in a silicon photonic platform^298^, as shown in Fig. 9b. The light coupling within silicon nanowaveguides tightly confines light at a subwavelength scale, thereby greatly enhancing the ORP and enabling the sorting of dielectric nanoparticles.

Technically, the separation of nanoparticles in a single light beam by trapping them at different positions is a technique known as “optical chromatography”^55,56^. This method seeks the balance of the ORP and extra forces, e.g., the gravity and fluidic drag force^135,299,300^. Notably, the levitation of particles enables various potential quantum applications^48,301^, such as quantum computing, sensing, optomechanics, etc. Applying a fluidic drag force is the most common way in optical chromatography, for instance, Zhu et al. configured a plasmonic microlens to realize label-free optical sorting of submicron exosomes^302^, as shown in Fig. 9c. This design has a minimal footprint of 4 × 4 µm^2^, making it feasible for practical on-chip applications.

In principle, harnessing optical stiffness plays an essential role in optical tweezers. For example, trapping of tiny bioparticles, such as viruses and proteins, requires a significant optical stiffness ranging from 10^−^^6^ to 10^−^^4^ N m^−1^ in order to generate sufficient OGFs^303–305^. Due to Brownian motion, nanoparticles oscillate within the potential well, exhibiting a mean square displacement (MSD) that describes the extent of oscillation. For an underdamped system, the MSD can be obtained by solving Eq. (21) as^7,306^

31 $${\rm{MSD}}(t)=\frac{2{k}_{B}T}{m{\omega }_{0}^{2}}\left[1-{e}^{-t/2{\tau }_{p}}\left(\cos {\omega }_{1}t+\frac{\sin {\omega }_{1}t}{2{\omega }_{1}{\tau }_{p}}\right)\right]$$

where *k*_*B*_ is the Boltzmann constant, *T* is the temperature, *m* is the mass of the particle; ${\omega }_{0}=\sqrt{k/m}$ is the resonant frequency with the trapping stiffness *k*; ${\tau }_{p}=m/(6\pi \eta a)$ is the momentum relaxation time of a particle with mass *m*; ${\omega }_{1}=\sqrt{{\omega }_{0}^{2}-{(1/2{\tau }_{p})}^{2}}$ is the corner frequency. In contrast, the MSD in an overdamped system can be expressed as

32 $${\rm{MSD}}(t)=\frac{1}{6\pi m\eta a{\omega }_{0}^{2}}\left[1-\frac{1}{2|{\omega }_{1}|{\psi }_{+}}{e}^{-t/{\psi }_{-}}+\frac{1}{2|{\omega }_{1}|{\psi }_{-}}{e}^{-t/{\psi }_{+}}\right]$$

where $${\psi }_{\pm }=2{\tau }_{p}/(1\pm 2{\tau }_{p}|{\omega }_{1}|)$$.

The oscillation range of the particle can be estimated from the square root of MSD, that is $\sqrt{{\rm{MSD}}}$. For a trapping system with a stiffness ranging from 10^−6^ to 10^−4^ N m^−1^, $\sqrt{{\rm{MSD}}}$ is normally smaller than 100 nm^307^, meaning that the particle is confined in a constrained space. Conventional optical chromatography typically employs a Gaussian beam, which rapidly diverges in intensity when tightly focused (a high stiffness). The rapid divergence in the direction of light propagation renders it unsuitable for the separation of nanoparticles. Consequently, an optical stiffness ranging from 10^−8^ to 10^−6^ N m^−1^ is typically employed in most optical chromatography configurations that utilize mostly Gaussian beams from a lens^56,308,309^, optical fibre^7,310^, etc.

Due to the contradictory nature of a Gaussian beam, which cannot exhibit both tight focus and slow divergence, conventional chromatography is typically suitable for microparticles. Therefore, the exploration of special light beams is necessary to address the specific requirements of manipulating nanoparticles. To push the boundaries of optical chromatography in terms of size and resolution, our group previously explored the optical sorting diagram and investigated the MSD in relation to the trapping stiffness and fluidic viscosity^7^. Ultimately, we identified a loosely overdamped system with extremely low stiffness (10^−10^ – 10^−8^ N m^−1^) that can be utilized for nanoparticle sorting. In the experiment, we successfully separated gold nanoparticles with radii ranging from 35 to 50 nm, achieving a resolution of 5 nm, as shown in Fig. 9d. Meanwhile, oscillation ranges of particles were measured to be several micrometers, which were significantly larger compared with previous optical tweezing systems. This system can also be utilized for the separation of nanosized bioparticles, such as bacteria and viruses. In addition to size, optical chromatography can also be employed to sort particles with varying refractive indices^56,311^.

### Sorting by the optical gradient force

OGFs can be used to manipulate and sort nanoparticles and cells. Sorting is accomplished by tailoring specific light waves and exposing particles to distinct force fields. The OGF can be positive and negative, conservative and nonconservative based on different sizes and refractive indices.

**Fig. 10:**
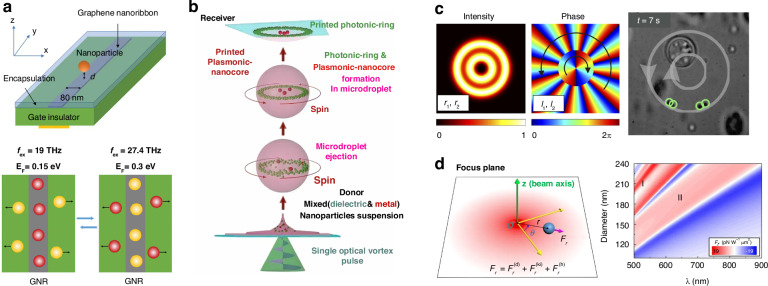
Sorting by the optical gradient force. **a** Manipulation of the polarization rate of particles is achieved by adjusting the excitation frequency, enabling the capture or rejection of Al_2_O_3_ nanoparticles and Fe_2_O_3_ nanoparticles by OGFs. Reproduced with permission^312^. Copyright 2021, Wiley-VCH GmbH. **b** The vortex beam traps polystyrene particles inside the outer ring and concentrates gold particles near the centre due to their positive and negative polarization rates. Reproduced with permission^313^. Copyright 2021, De Gruyter under CC BY 4.0. **c** Yeast cells and silica spheres of different diameters are sorted based on their response to the OGF exerted by the inner and outer rings. Reproduced with permission^314^. Copyright 2021, Optical Society of America. **d** Particles with sizes between the first and second Kerker conditions can be separated using nonconservative OGFs. Reproduced with permission^315^. Copyright 2020, WILEY-VCH Verlag GmbH & Co. KGaA

Equations (1)–(3) show that the intensity gradient force or the OGF is linked to the real part of the particle polarizability, which varies positively or negatively depending on the size of the metallic (e.g., gold) nanoparticles and the light wavelength. This is also due to the coordination of the wavelength-dependent real and imaginary parts of the permittivity of metallic nanoparticles. Based on this principle, Au nanoparticles with varying sizes can be sorted^117^. Paul and Liu configured graphene plasmonic optical tweezers working at the mid-infrared range^312^, as shown in Fig. 10a. Within this wavelength range, nanoparticles like Al_2_O_3_, Fe_2_O_3_ and SiO_2_ may exhibit distinct polarizabilities at specific wavelengths, resulting in distinct OGFs that lead to the selective trapping and repulsion of nanoparticles. Due to opposite polarizabilities [cf., Eqs. (1) and (2)], dielectric and metallic nanoparticles could experience attractive and repulsive OGFs, respectively. Thus, by utilizing a single optical vortex pulse, Kawaguchi et al. trapped polystyrene nanoparticles in an outer ring while confined gold nanoparticles in the inside core^313^, as shown in Fig. 10b. Figure 10c shows a special light-field structure designed by Bobkova et al., which attracts larger particles by the dominant large OGFs in the inner ring^314^. As a result, larger particles aggregate in the inner ring and move clockwise. On the other hand, smaller particles, due to their small dimensions, only undergo intensity gradient forces in the outer ring and rotate anticlockwise along the outer ring. This process sorts yeast cells and silica spheres of different diameters.

In optical tweezers, the OGF is typically considered conserved and has a uniform directionality^45^. However, Xu et al. showed that in optical tweezers made up of linear or elliptically polarized Gaussian beams, the OGF generated by the Kerker interference is nonconservative and has a directional asymmetry that repels particles away from the beam axis^315^. Sorting is achieved by utilizing the Kerker-Type intensity-gradient force to repel particles with sizes falling between the first and second Kerker conditions, as illustrated in Fig. 10d. By leveraging this force-inverse effect, this technique can achieve a sorting resolution of less than 5 nm. The OGF can also interact synergistically with the ORP in an optical woven spiral beam, enabling the trapping of particles of various sizes at different junctions^316^.

Using movable unwound polygonal vortex beams, Li et al. were able to sort nanoparticles massively and efficiently^317^. Nanoparticles with higher refractive indices or larger sizes are more likely to be attracted and pulled by the locomotive OGF, allowing them to be separated from particles with lower refractive indices or smaller sizes. Similarly, one can leverage various strategies involving moving potential wells for optical sorting, such as employing a monolayer conveyor^318^.

An exemplary and well-defined optical sorting strategy is “holographic optical tweezers,”^319–322^, which utilize an optical lattice with focused laser spots to create OGFs on particles. Holographic optical tweezers can be used for multifunctional and three-dimensional manipulation of particles in a still environment^320,322–324^, offering greater flexibility and control compared to traditional single-beam optical tweezers. Meanwhile, when subjected to a flow, the OGF can coordinate with the fluid drag force to divert particles from their original flow direction. The deviation angle may vary for different particles due to size-dependent optical and fluidic forces, thereby achieving the sorting function.

**Fig. 11:**
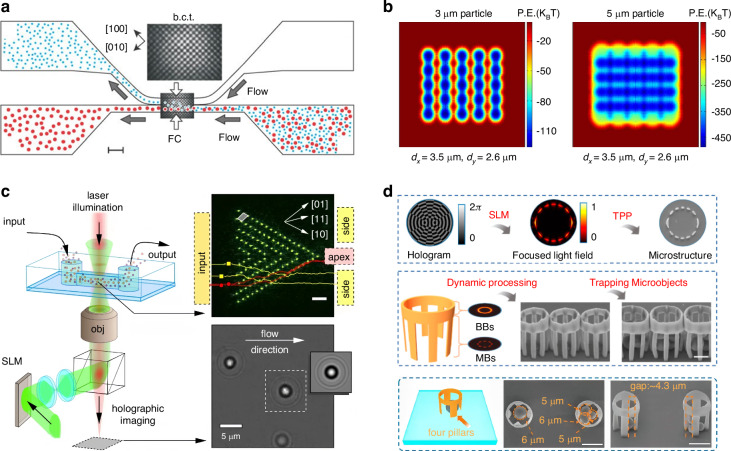
Holographic optical tweezers for particle sorting based on the optical gradient force. **a** Utilizing holographic optical tweezers for efficient sorting of micron-sized particles. Reproduced with permission^325^. Copyright 2003, Springer Nature. **b** Using holography to create optical traps in orthogonal directions for experimental sorting of 3 μm and 5 μm silica microspheres. Reproduced with permission^328^. Copyright 2012, IOP Publishing. **c** Sorting and tracking colloidal particles with varying sizes and refractive indices in an oblique lattice. Reproduced with permission^329^. Copyright 2010, American Physical Society. **d** The holographic construction of Mathieu beams is employed for capturing and sorting silica microparticles of varying sizes through the creation of microcages. Reproduced with permission^330^. Copyright 2019, American Chemical Society

The pioneering attempt to utilize holographic optical tweezers for optical sorting was made by MacDonald et al. 20 years ago^325^. They configured a dynamically reconfigurable, three-dimensional optical lattice, where particles with different polarizabilities experience different OGFs, thus enabling a tunable sorting criterion with the assistance of a flow, as shown in Fig. 11a. Sorting by size and refractive index can be achieved with an efficiency approaching 100%. This work opens a new avenue for sorting particles with holographic optical tweezers. Shortly thereafter, Ladavac et al. implemented an array of hotspots using holographic tweezers and studied the selectively locking effect of particles with varying sizes^326^. By designing closed-loop holographic optical tweezers, Chapin et al. achieved the multifunctional manipulation of particles, such as trapping, assembly and sorting^327^. It is demonstrated that 1 µm and 1.9 µm silica particles can be efficiently separated by assigning distinct trajectories to different particles. In the optical lattice, the potential well depths for silica particles of different sizes, such as 3 µm and 5 µm, can be dynamically adjusted, allowing them to move in an orthogonal direction within the optical lattice^328^, as shown in Fig. 11b.

Additional optical lattice configurations, such as the oblique setup, could provide a more precise and powerful tool for optically sorting particles based on not only different sizes but also different refractive indices^329^, as shown in Fig. 11c. Advanced optical patterns could facilitate more intricate multidimensional sorting, offering significant advantages for medical diagnostics. Recently, utilizing holographic techniques, Wang et al. generated Mathieu beams and Bessel beams to create a three-dimensional microcage, showing great promise in isolating particles of different sizes^330^, as shown in Fig. 11d. This rapid and advanced fabrication method offers a novel approach to particle sorting, isolation and detection.

Holographic optical tweezers are typically accompanied by zero-order diffraction^331,332^, which can occasionally disrupt the optical manipulation. To solve this issue, Zhang et al. proposed a zero-order free holographic apparatus by virtue of an asymmetric triangle reflector and a digital lens^333^. This architecture will greatly benefit three-dimensional optical manipulation, particularly in efficiently sorting particles. Holographic optical tweezers can also work in the near field, for instance, holographic surface-wave tweezers^334^. They can also work at the interface of air and liquid^335^, and be integrated with other techniques, such as Raman spectroscopy^283,336,337^, to enhance sensitivity and effectiveness in manipulation.

### Sorting by the phase-gradient force

The phase-gradient force was first systematically formulated and studied by Roichman et al. in 2008^61^, while the first demonstration was carried out by O’Neil et al. who observed the particle dynamics in the ring-shaped cross-section of a Laguerre–Gaussian light beam in 2002^338^. In essence, according to Eqs. (3) and (7), the phase-gradient force is a type of force arising from the orbital momentum, ${{\bf{p}}}^{O}$. Therefore, similar to the ORP, the phase-gradient force is sensitive to the size and refractive index of the particle, forming the basis for optical sorting utilizing this force.

**Fig. 12:**
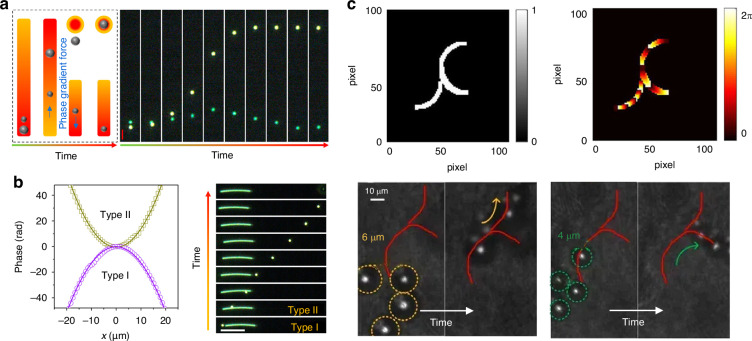
Sorting based on the phase-gradient force. **a** Sorting of gold and silver nanoparticles based on competition between phase-gradient force and intensity-gradient force. Reproduced with permission^64^. Copyright 2018, American Chemical Society. **b** Separation of Ag nanowires with varying sizes by designing a phase-gradient optical field. Reproduced with permission^339^. Copyright 2020, American Chemical Society. **c** Particle sorting in an optical junction with a phase gradient. Reproduced with permission^340^. Copyright 2021, AIP Publishing

Recently, Nan and Yan have thoroughly investigated optical sorting using the phase-gradient force. For example, through the precise control of phase distributions of line-shaped beams, metallic nanoparticles of varying sizes will experience unique phase-gradient forces, resulting in distinct velocities of movement^64^, as shown in Fig. 12a. They can also control temporal and spatial distributions of phase, achieving a superb sorting performance with sub-100 nm size and 10 nm resolution.

The phase-gradient force can also combine extra forces such as the OGF and fluidic drag force to enhance the efficiency of optical sorting. Nan and Yan utilized the fluidic drag force to transport nanoparticles and to balance the phase-gradient force^63^. Akin to the optical chromatography, equilibrium positions for sub-50 nm gold nanoparticles with various sizes are different, resulting in a sorting resolution of 1 nm. The same group later extended this technique to explore the synergy of intensity, phase and polarization on the effect of optical sorting^65^. Various properties of light result in different forces, such as the OGF, phase-gradient force, and more, all working together to achieve exceptional sorting performance. By delicately controlling different properties of light, the Ag nanowire (70 nm diameter and 6 µm length) can be separated from the 150 nm Ag nanosphere (Fig. 12b)^339^. Meanwhile, using this approach, nanoparticles can be driven to move in complex two-dimensional trajectories. Following a similar principle that utilizes the OGF and phase-gradient force, Zhou et al. proposed a nontrivial optical junction capable of sorting particles of different sizes by directing them towards different branches^340^, as shown in Fig. 12c. The utilization of the phase-gradient force introduces a new level of control for optical sorting. This force can be used independently or in conjunction with other types of forces to achieve more precise and robust sorting.

### Sorting by the optical pulling force

**Fig. 13:**
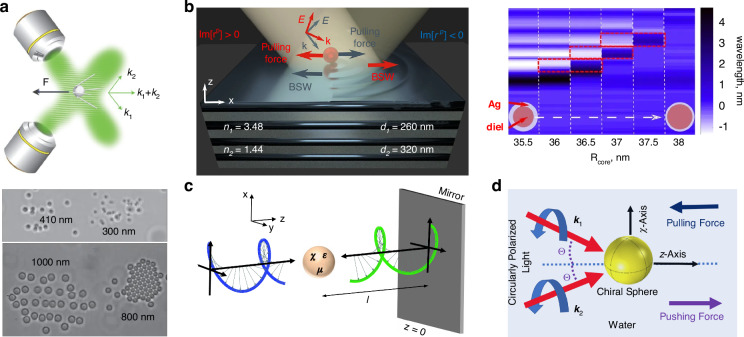
Sorting by the optical pulling force. **a** Separating 300 nm & 410 nm, 800 nm & 1000 nm polystyrene particles by controlling light incident angles and polarizations of light beams. Reproduced with permission^66^. Copyright 2013, Springer Nature. **b** A one-dimensional photonic crystal is capable of producing a Bloch surface wave to generate the OPF, enabling the sorting based on the resonance position of the particle. Reproduced with permission^342^. Copyright 2022, Optica Publishing Group. **c** Optical sorting of chiral particles in an optical conveyor belt using an unstructured chiral lightwave. Reproduced with permission^345^. Copyright 2016, American Physical Society. **d** Optical sorting of chiral particles using the OPF in a two-wave interference optical field. Reproduced with permission^346^. Copyright 2021, Optical Society of America

The OPF offers an efficient alternative tool for optical sorting by reversing the optical force from positive to negative^341^. The experimental demonstration of the OPF was done by O. Brzobohatý et al. in 2013 using a Bessel beam generated by two interference lightwaves^66^, as shown in Fig. 13a. Various polarizations have been found to have a significant influence on the OPF, which is also employed for sorting particles based on their sizes. By switching the light polarization and controlling incident angles of two interfering beams, 600 nm and 820 nm polystyrene particles can be separated.

The OPF can occur in the near field in Bloch surface waves^342^. It emerges on a single dipolar particle above a one-dimensional photonic crystal owing to the directional excitation of Bloch surface waves. The OPF can be tailored by altering the wavelength and incident angle, and can be utilized to sort particles based on different sizes and compositions (e.g., core-shell structure)^342^, as shown in Fig. 13b. In a topological environment characterized by a unique momentum topology, the shape of particle could significantly impact the forward momentum and give rise to the OPF^343^. As a result, the momentum-topology-induced OPF shows some potential for shape-selective sorting.

Apart from achiral particles, chiral particles can also be sorted by the OPF^344^. Fernandes et al. configured an optical conveyor belt using an unstructured chiral lightwave^345^. Chiral particles can either be pushed or pulled depending on their chiral responses, as illustrated in Fig. 13c. Ali et al. calculated optical forces on chiral particles inside two-collinear, circularly polarized light beams. They discovered that the OPF occurs at different light polarizations, incident angles, as well as correlates closely with the particle size and chirality parameter. This highlights the significant potential of OPF in sorting microspheres^346^, as depicted in Fig. 13d.

### Sorting by the optical lateral force

**Fig. 14:**
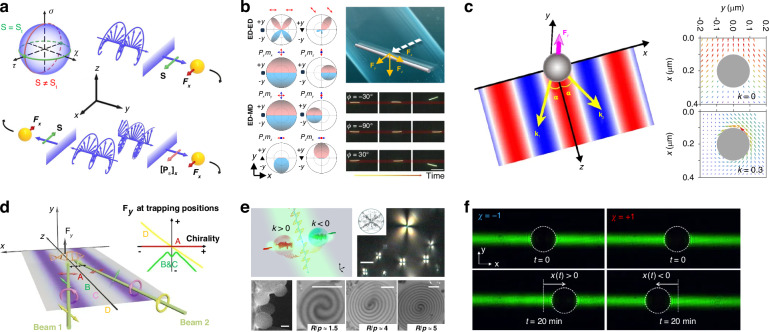
Optical sorting by the optical lateral force. **a** OLFs on an achiral particle in an evanescent wave that carries pure transverse spins. Reproduced with permission^347^. Copyright 2023, Wiley-VCH GmbH. **b** OLF on a nanowire under the illumination of a linearly polarized beam. Reproduced with permission^351^. Copyright 2023, Springer Nature. **c** Sorting of chiral particles in the interference field configured by two linearly polarized plane waves. Reproduced with permission^353^. Copyright 2016, American Physical Society. **d** All-optical sorting of chiral particles in a standing wave by two linearly/circularly polarized light beams. Reproduced with permission^354^. Copyright 2017, American Chemical Society. **e** Experimental demonstration of sorting of chiral microparticles by the linear momentum transfer. Reproduced with permission^72^. Copyright 2020, Springer Nature. **f** Employing the Optical Stern-Gerlach Newton experiment for sorting different-handed chiral particles with a diameter of 30 µm. Reproduced with permission^361^. Copyright 2019, American Physical Society

We have shown that chiral particles can be sorted using the OGF, ORP and OPF, we hereby reveal that the OLF provides an alternatively efficient tool for enantioselective sorting. According to Eq. (10), the OLF on an achiral dipole sphere can originate from the curl of the spin angular momentum, $\nabla \times {\bf{S}}$, which is called the Belinfante spin momentum^85^. In this case, the OLF is proportional to $\mathrm{Re}({\alpha }_{ee}{\alpha }_{mm}^{\ast }){{\bf{p}}}_{S}^{m}$, where^85^

33 $${\mathrm{Re}}({\alpha }_{e}{\alpha }_{m}^{\ast })\cong \frac{{k}^{2}{a}^{8}}{30}{|\frac{{\varepsilon }_{p}-\varepsilon }{{\varepsilon }_{p}+2\varepsilon }|}^{2}[{\mathrm{Re}}({\varepsilon }_{p})+2]$$

where *ε*_*p*_ is the permittivity of particle. Since $\mathrm{Re}({\varepsilon }_{p})+2 \,> \,0$ and could possibly <0 for dielectric and metallic nanoparticles at 532 nm, respectively, the OLF from the Belinfante spin momentum can be utilized for bilaterally sorting those two types of particles. Using the multipole expansion theory, Yu et al. found that the achiral particle in an evanescent wave that carries pure transverse spin can experience an OLF from the spin^347^, as shown in Fig. 14a. The OLF in this system correlates tightly with the particle size, potentially producing the reversible OLF and demonstrating the feasibility of this method in optical sorting.

The OLF is normally accompanied by exotic materials and/or complex lightwaves^348–350^. Recently, Nan et al. reported a nontrivial OLF on a nanowire under the illumination of a linearly polarized beam^351^, as shown in Fig. 14b. The underlying physics hinges on the multipolar interplay of the particle. Following thorough calculations, the sign of OLF is linked to the size and length-radius ratio of the nanowire, highlighting the potential of this method in shape-based optical sorting.

It has been manifested that the OLF is particularly effective for enantioselective sorting^352^, as the chiral OLF can reverse its sign at opposite chirality parameters *κ*. Various strategies have been proposed to harness the OLF for sorting chiral particles. For instance, chiral particles on a surface experience reversible OLFs under the illumination of a linearly polarized beam^69^. Optical sorting of chiral particles can also be accomplished in a standing wave created by two linearly polarized beams, where the OLF arises from the transverse spin and energy vortex^353^, as shown in Fig. 14c. When two light beams forming the standing wave are circularly polarized, additional components for OLF emerge, potentially enhancing the feasibility of optical sorting^354^, as shown in Fig. 14d. Theoretically, chiral sorting using the OLF can happen in evanescent waves^352^, vector beams^355^, surface-plasmon-polariton systems^356,357^, as well as many other platforms^358–360^.

Most of those works are theoretical investigations. In the experimental exploration, Shi et al. synthesized chiral microparticles and placed them at the interface of air and water, finding that the OLF emerges by the lateral momentum transfer between different media^72^, as illustrated in Fig. 13e. Notably, the OLF not only reverses its sign with the chirality parameter but also with the particle size, and light polarization. Thus, they realized the bidirectional sorting of chiral particles with different handedness. Kravets et al. demonstrated the optical Stern-Gerlach experiment where the chiral particles can be sorted by OLFs from optical helicity gradients^361^, as shown in Fig. 13f. Exploiting the legacy of the Stern-Gerlach experiment in the realm of chiral light-matter interactions should encourage additional researches, such as the development of chirality-enabled quantum technologies or spin-based optoelectronics.

Chiral sorting with the OLF offers several advantages, including high precision, dynamism, and efficiency. However, there are also inherent weaknesses that require improvement, such as low throughput and large particle size. The development of a rapid and efficient platform for sorting nanosized chiral particles deserves future thorough investigations.

### Control of potential wells

The integral of force with the displacement gives rise to the formation of the optical potential well. For a particle in a Cartesian coordinate, its potential energy *U* at (*x*, *y*) can be expressed as^134,362,363^

34 $$U(x,y)={\int_{0}^{y}}F(y,0)\,dy+{\int_{0}^{x}}F(y,x)\,dx$$

where *F*(*y*, 0) and *F*(*y*, *x*) denote optical forces at (*y*, 0) and (*y*, *x*), respectively. From Eq. (34), it is evident that the potential energy is related to the optical force and the integral path. Thus, it increases linearly with the optical stiffness. Since the optical force is size- and shape-dependent, different particles may have different depths of potential well (potential energy). For stable trapping, the potential well depth *U* should satisfy |*U* | >10*k*_*B*_*T* (empirical formula)^45,134,364^, where *k*_*B*_ is the Boltzmann constant, *T* is the temperature. One efficient strategy for optical sorting involves adjusting the laser power to control the depths of various potential wells, ensuring that the potential energy (*U*) of small particles is less than 10*k*_*B*_*T* while that of larger particles is greater than 10*k*_*B*_*T*. As a result, the big particle will be trapped, while the small one will be released.

**Fig. 15:**
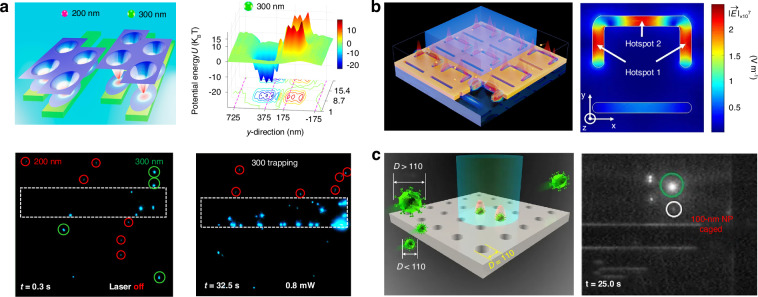
Sorting of spherical particles by adjusting depths of potential wells. **a** Massive sorting of 200 – 500 nm nanoparticles in coupled hotspots in a nanowaveguide array. Reproduced with permission^362^. Copyright 2020, American Chemical Society. **b** Sorting of nanoparticles with a plasmonic optical tweezer based on the self-induced back-action effect and Fano resonance. Reproduced with permission^365^. Copyright 2023, American Chemical Society under CC BY 4.0. **c** Sorting of single viruses using a dielectric nanocavity array. Viruses of different sizes can be confined within nanoholes of specific diameters. Reproduced with permission^59^. Copyright 2022, Wiley-VCH GmbH

One example is the control of potential well in paired nanowaveguide array^362^, as shown in Fig. 15a. The coupling of light between two adjacent nanowaveguides creates isolated hotspots with a size determined by the width of the nanowaveguide, overcoming the diffraction limit. By precisely controlling the potential well depth, 200-nm polystyrene nanoparticles can be separated from 300 nm and 500 nm ones; 300 nm polystyrene nanoparticles can be separated from 500 nm ones. The sorting efficiency can approach 100% by designing a microfluidic chip with a height less than 1 µm, ensuring that nanoparticles are within the near-field range, typically only a few hundred nanometers. This sorting methodology can be applied to plasmonic optical tweezers to increase the trapping stiffness of nanoparticles. For instance, Bouloumis et al. recently presented a metamaterial plasmonic tweezers capable of achieving a trapping stiffness as high as 4.48 ± 0.2 (fN nm^−^^1^)(mW µm^−2^) for the 20 nm gold nanoparticle^365^, as shown in Fig. 15b. This device can also be deployed for selectively trapping different bioparticles as stated by the authors. Significantly, the authors of this study explored the intriguing self-induced back-action effect, wherein the presence of nanoparticles could significantly impact the trapping light field, modifying the trapping stiffness and occasionally even reversing the direction of the optical force^366–368^. The use of plasmonic optical tweezers for trapping inevitably induces a heating effect that may be harmful to bioparticles. To solve this issue, dielectric nanocavities are proposed^369^, which generate large optical forces with Mie resonances^59^, as shown in Fig. 15c. Nanoholes with diameters approximately 100 nm trap light inside, resulting in a substantial enhancement of OGF for trapping individual viruses smaller than the nanohole, while keeping larger viruses outside of the nanohole. Successively, larger viruses would be flushed away by the flow stream when the laser is turned off. This method has a high resolution in screening single viruses using nanoholes with different diameters. It also provides a versatile tool for multifunctional manipulation of single viruses, such as sorting, transporting, trapping and detection ^59,370,371^.

**Fig. 16:**
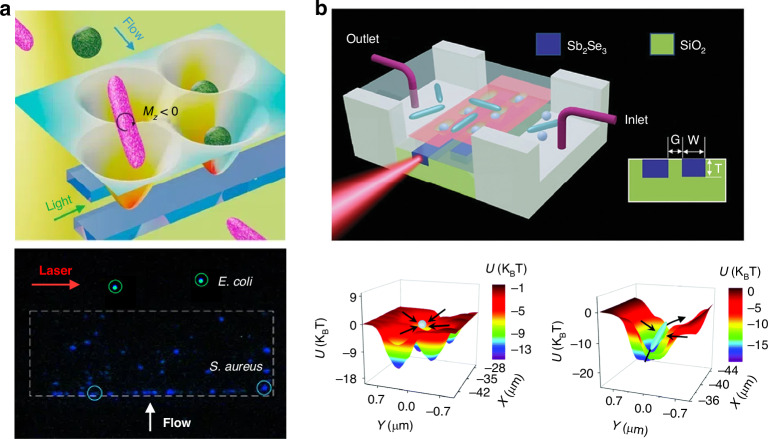
Shape-selective sieving using potential well arrays. **a** Nanophotonic sawtooth array for the separation of rod-shaped *E*. *coli* and spherical *S*. *aureus* by exploiting their different dynamic behaviours in coupled potential wells. The separation efficiency can reach 95%. Reproduced with permission^372^. Copyright 2019, American Chemical Society. **b** Selective trapping and transporting of different-shaped particles by adjusting the transition of Sb_2_Se_3_ between amorphous and crystalline phases, which modifies the coupling length between hot spots. Reproduced with permission^373^. Copyright 2022, Royal Society of Chemistry

Aside from spherical nanoparticles, the optical potential well array has the capability to sort rod-shaped particles. Figure 16a depicts the separation of two types of bacteria: spherical *S. aureus* and rod-shaped *E. coli*. ^372^. The sorting is realized by exploiting the distinct dynamic behaviours of different shaped particles. The spherical *S. aureus* can be trapped inside potential wells, while rod-shaped *E. coli* twists and is transported between potential wells, consequently being released. The sorting efficiency reaches 95% given a suitable flow velocity and laser power. A similar idea was conducted by Cao et al., who utilized optofluidic nanophotonic paired waveguides composed of chalcogenide semiconductor Sb_2_Se_3_ to create coupled hotspots^373^, as shown in Fig. 16b. The coupling length can be actively tuned via the transition of Sb_2_Se_3_ between amorphous and crystalline phases, offering a dynamic control of particle behaviours inside the system.

Examples for sorting rod-shaped particles presented above have a limitation in the manipulation speed. This is due to the short range of near field, and relatively low laser powers in nanowaveguides. A promising approach to enhance the sorting speed is to leverage the advantages of flow cytometry and machine learning algorithms. This aspect will be discussed in Outlook section.

**Fig. 17:**
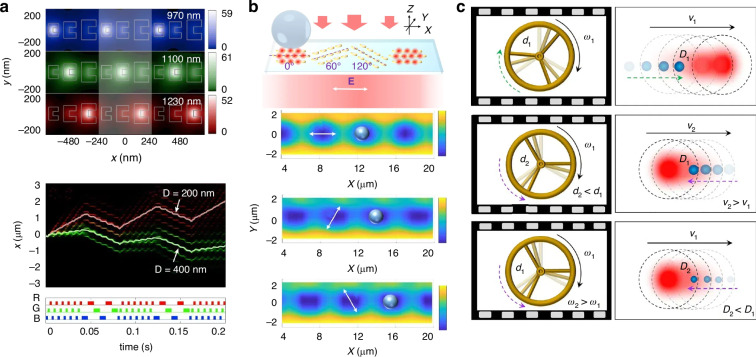
Regulating the motion of potential wells for optical sorting. **a** Optical conveyor belt consists of three substructures for sorting 200 nm and 400 nm magnetic particles. Reproduced with permission^376^. Copyright 2014, American Chemical Society. **b** Plasmonic metasurface enabled optical conveyor for sorting particles with sizes of 2 µm, 3 µm, and 4 µm by adjusting different polarizations of light. Reproduced with permission^380^. Copyright 2021, Optical Society of America. **c** Optical wagon-wheel effect to achieve the separation of 102 nm and 155 nm polystyrene nanoparticles. Reproduced with permission^381^. Copyright 2021, Wiley-VCH GmbH

Sometimes, potential wells are not stationary and can be mobile, a feature that can also be utilized for optical sorting^374,375^. In this scenario, optical sorting relies on the fact that different particles possess distinct potential well depths and optical forces, resulting in varying velocities when the potential well moves in a specific direction or undergoes periodic modulations. These different particle velocities ultimately lead to their separation. One noteworthy example is the optical conveyor belt^376–379^, which regulates the movement of potential wells by employing various resonance wavelengths. Figure 17a illustrates the theoretical demonstration of sorting 200 nm and 400 nm nanoparticles using a nano-optical conveyor belt, comprising three substructures under the resonance of three different wavelengths within a single unit cell^376^. When the wavelength is repetitively varied, the position of the potential well changes, causing particles to move at different velocities. In the experiment, Zheng et al. designed three substructures under the resonance of three different polarization angles^377^, and demonstrated the sorting of polystyrene nanoparticles ranging in size from 200 nm to 500 nm.

The conveyor belt can be configured with plasmonic metasurface^380^, as shown in Fig. 17b. Gold plasmonic elliptical elements can be excited to generate different hotspots using different polarizations. This design only requires a minimal laser intensity of 0.08 mW µm^−2^ to obtain a potential well depth as high as 10 *k*_*B*_*T*, and can be used for various on-chip optofluidic applications. Xu et al. explored the optomechanical wagon-wheel effect for the directional sorting of polystyrene nanoparticles^381^, as shown in Fig. 17c. When a laser beam is scanned at different frequencies, nanoparticles with different sizes are moving at different directions and with different velocities, by analogy with the wagon-wheel effect. They experimentally achieve sorting of polystyrene nanoparticles down to the sub-200 nm scale.

### Optothermal tweezers

The heating effect is typically a detrimental phenomenon in optical tweezers that people have long sought to avoid^45,382–385^. The pronounced Brownian motion in the potential well renders the trapping unstable as the temperature is raised. This situation changes when heating is used for optical manipulation, leading to the emergence of optothermal tweezers^382,386–393^. In 2010, Liu et al. found that the heating of water could induce a fluid drag force capable of attracting particles in a long range, demonstrating the versatile manipulation of particles using optothermal tweezers with a single laser beam^394^. Similarly strategy was later deployed in the near field in 2015 when Ndukaife et al. developed a hybrid electro-thermo-plasmonic nanotweezer capable of achieving the long-range and rapid transport of nanoparticles^395^.

**Fig. 18:**
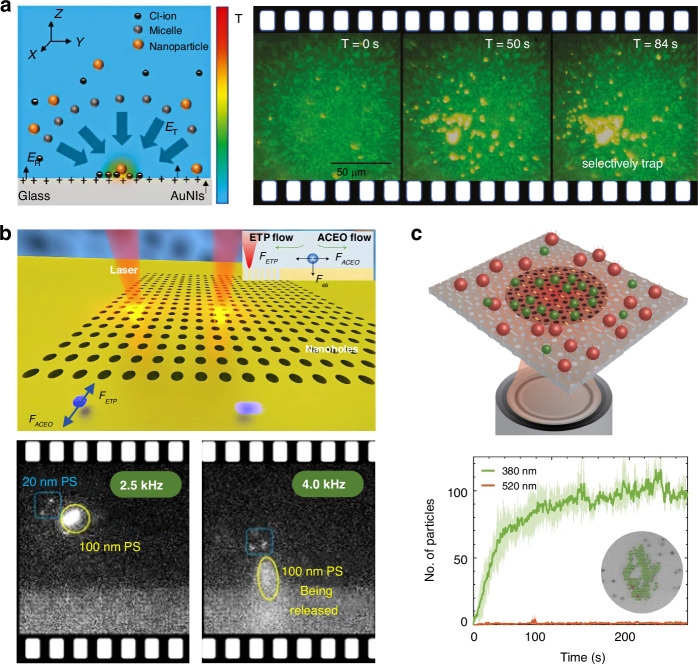
Optical sorting using optothermal tweezers. **a** Selective trapping of particles by opto-thermoelectric speckle tweezers. Reproduced with permission^396^. Copyright 2020, De Gruyter under CC BY 4.0. **b** Opto-thermo-electrohydrodynamic tweezers for the separation of 20 nm and 100 nm polystyrene nanoparticles. Reproduced with permission^58^. Copyright 2020, Springer Nature. **c** Adjusting selective trapping by varying the surfactant concentration in the solution to sort 380 nm and 520 nm polystyrene nanoparticles. Reproduced with permission^397^. Copyright 2018, Optical Society of America

For the purpose of optical sorting, Kotnala et al. developed an opto-thermoelectric speckle tweezers that utilize OGFs from randomly distributed laser spots^396^, as shown in Fig. 18a. The hotspots induce OGFs and thermoelectric forces on particles for the trapping. When a flow is applied, the optical and thermoelectric forces trap 200 nm particles onto the speckle, while they are unable to overcome the fluidic drag force for 1 µm particles, resulting in their escape. By switching the frequency of the opto-thermo-electrohydrodynamic tweezers, Hong et al. were able to trap 20 nm polystyrene nanoparticles within the plasmonic nanohole array, while sorted out 100 nm ones^58^, as shown in Fig. 18b. This technique serves as a paradigm for sorting of sub-100 nm bioparticles, such as proteins and exosomes. Krishnan et al. discovered that by altering the concentration of surfactant, the strength of the thermophoretic effect can be modified, which in turn affects the size selectivity of captured particles^397^, as shown in Fig. 18c.

Optothermal tweezers, which expand the trapping range and enhance the trapping speed of nanoparticles, are expected to play increasingly significant roles in efficient optical sorting.

### Field enhancement for optical sorting

Enhancement of the optical field is a straightforward way to enlarge the optical force and improve the sorting efficiency. There are a great diversity of methods for the field enhancement, for instance, SPR^398–402^, Fano resonance^365,403–405^, quasi-BIC^16,406–410^, ring resonantor^411–414^, nanocavities^305,415–417^, multipoles^16,418–421^, etc.

**Fig. 19:**
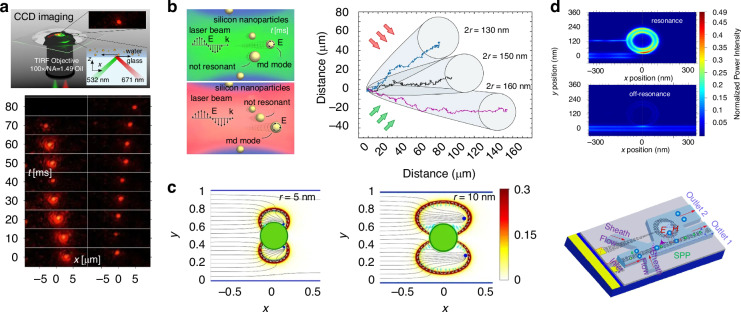
Optical sorting with the field enhancement. **a** Bidirectional sorting of single gold nanoparticles with surface plasmon resonances (SPRs) using two counter-propagating evanescent waves. Reproduced with permission^422^. Copyright 2012, American Chemical Society. **b** Sorting of silicon nanoparticles by exploring the Mie resonance. Reproduced with permission^67^. Copyright 2017, American Chemical Society. **c** Self-trapping of particles of different sizes using a quasi-BIC in a waveguide cavity. Reproduced with permission^427^. Copyright 2022, American Physical Society. **d** Enhancing OGF for optical sorting using a ring resonator. Reproduced with permission^428^. Copyright 2019, Optical Society of America

For the sorting purpose, Ploschner et al. excited two counter-propagating evanescent waves to sort two gold nanoparticles with different sizes^422^, as shown in Fig. 19a. 100 nm and 150 nm gold nanoparticles exhibit SPRs near 532 nm and 671 nm, respectively, enabling their separation via ORPs from distinct light sources. By harnessing high-quality Mie resonance, Shilkin et al. demonstrated the sorting of silicon nanoparticles of various sizes, i.e., 130, 150 and 160 nm^67^, as shown in Fig. 19b. The approach involves exciting various optical modes on nanoparticles, including electric and magnetic dipoles and quadrupoles, which have a significant impact on the ORPs exerted on them. BIC is a unique and counterintuitive phenomenon in physics where a bound state exists within the continuum spectrum of modes in a system, which has great potential for optical sensing and particle manipulation^13,423–426^. Bulgakov et al. recently integrated cylinders into a metal waveguide to form a Fabry-Perot resonator^427^. They utilized a quasi-BIC to achieve self-trapping of particles of different sizes. Particles with radii of 5 and 10 nm were captured at different locations to achieve sorting of nanoparticles, as shown in Fig. 19c. Recently, Bulgakov and Sadreev^409^ employed the BIC in a dual finite grating to achieve precise sorting of sub-100 nm nanoparticles using a minimal laser intensity in the order of 1 mW µm^−2^.

The above examples show the enhancement of ORP for optical sorting, while the OGF can also be largely enhanced for sorting nanoparticles. As shown in Fig. 19d, Abbasi et al. modulated the refractive index of the micro-ring resonator by electro-optic or thermo-optic means, thereby altering the resonant state of the ring^428^. This change in state affects the distribution of the optical field in the coupling region between the ring resonator and the linear waveguide, thereby affecting the potential well. When the ring is in the on-resonant state, particles enter the ring, while particles flow out of the ring when it is in the off-resonant state. Using the microring resonator, polystyrene particles down to the size of 22 nm can be sorted.

### Metasurface-assisted optical sorting

Recent years have witnessed the emergence of optical manipulation with metasurfaces because of superb capabilities of metasurfaces in steering electromagnetic waves^34^. Metasurfaces is able to maneuver the amplitude, phase, momentum topology, and angular momentum of light to endow multifunctional manipulations of particles such as trapping, pulling, transporting and sorting^429–438^. Intriguingly, metasurfaces can also be peeled off from the substrate and float on the surface of air. They could move in complex trajectories under the illumination of light, forming the so-called “metarobots”. Herein, we only review how the metasurface can be used to sort particles in an efficient manner.

**Fig. 20:**
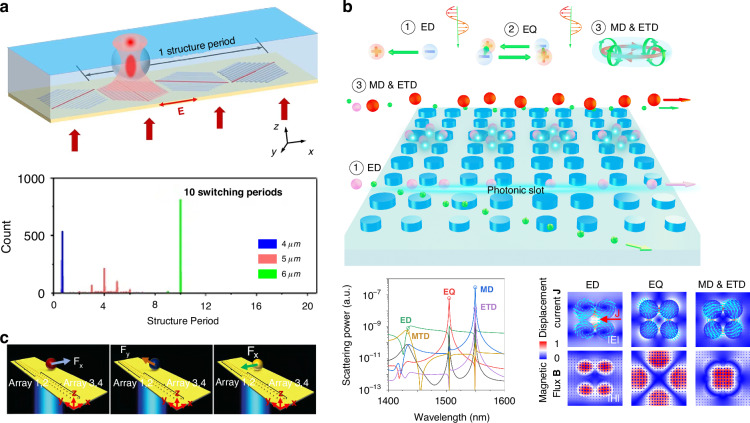
Metasurface-assisted optical sorting. **a** Levitated optical conveyor belt using the metasurface for the separation of 4 µm, 5 µm and 6 µm polystyrene particles. Reproduced with permission^439^. Copyright 2022, Optica Publishing Group. **b** Leveraging multipoles and toroidal dipoles to sort sub-10 nm polystyrene nanoparticles with a resolution of 1 nm. Reproduced with permission^440^. Copyright 2023, Wiley-VCH GmbH. **c** Sorting of dielectric, metallic, and chiral particles using various optical forces within a metasurface comprised of double-lined gold nanostructures. Reproduced with permission^358^. Copyright 2021, Wiley-VCH GmbH

Figure 20a shows an example of a levitated optical conveyor belt using the metasurface^439^. The conveyor belt is configured using a polarization-dependent nanoslit-based metasurface lens, whose resonance can be tuned using different polarizations, resulting in the shifting of potential wells. Consequently, 4 µm, 5 µm and 6 µm particles can be conveyed and sorted with a low laser power <1 mW µm^−2^.

To further explore the sorting limit in the speed and particle size, Luo et al. recently proposed a nontrivial all-dielectric metasurface that can generate multipoles for optical sorting^440^, as shown in Fig. 20b. It is manifested that electric dipole and quadrupole, as well as electric toroidal dipole, can all effectively confine light on a deep-subwavelength scale, enhancing the optical field and producing increased optical forces. The separation of 9 nm and 10 nm polystyrene nanoparticles has been successfully demonstrated, achieving a sorting resolution of 1 nm. Besides, 17 nm and 20 nm exosomes can be separated with a sorting resolution of 3 nm, which is superb for bioparticles. Significantly, velocities of exosomes can reach 300 µm s^−1^ using a minimal laser intensity of 9.5 mW µm^−2^. This optofluidic platform serves as a paradigm for the sorting of sub-20 nm bioparticles, such as exosomes and viruses.

Metasurfaces can also sort chiral particles, showing great advantages of optical approaches compared with previous chemical ones. Safkat et al. achieved the sorting of dielectric, metallic and chiral particles simultaneously using a metasurface composed of double-lined gold nanostructures^358^, as shown in Fig. 20c. This device generates a dual surface plasmon polariton energized plasmonic complex field, inducing different behaviours in Mie scatterers with dissimilar material properties. For instance, under the illumination of a plane wave, the dielectric particle experiences an ORP, the plasmonic particle experiences the counterintuitive OPF, whereas the chiral particle experiences an OLF, thus they can be separated efficiently.

Liu et al. proposed an asymmetric dielectric tetramer metasurface, which can separate enantiomers under the excitation of circularly polarized lightwaves^432^. The chiral optical force can push one enantiomer towards regions of the quasi-BIC system while eliminating the other with the opposite handedness. The symmetry-broken tetramer metasurface can provide multiple Fano resonances and deep trapping potential wells with depths up to 33 *k*_*B*_*T* with moderate laser power. This system is well feasible for trapping achiral particles and sorting chiral particles, having potential in some biological and physical applications.

It is worth noting that optical sorting using metamaterials is just in the nascent stage. More and more designs are flourishing rapidly with the emergence of new ideas in cutting-edge nanophotonic technologies. In the next section, we shall show a fascinating example of optical sorting using metasurfaces by exploiting intriguing topological physics.

### Optical sorting in topological optical fields

Topological optical field refers to a specialized area in optics that explores the topological properties of light fields^441–444^. So far, a great diversity of topological optical fields, e.g., structured light, are used for optical manipulation, such as optical vortices, phase singularities and other complex wavefront shapes^445–448^. It involves the manoeuvre of the spatial and spectral characteristics of light in relation to its underlying topological features. Topological optics are of interest for applications in optical communications and imaging^444,449,450^, quantum optics^451–453^, optical manipulation^131,454,455^, and many other disciplines where controlling topological properties of light is advantageous^456^.

**Fig. 21:**
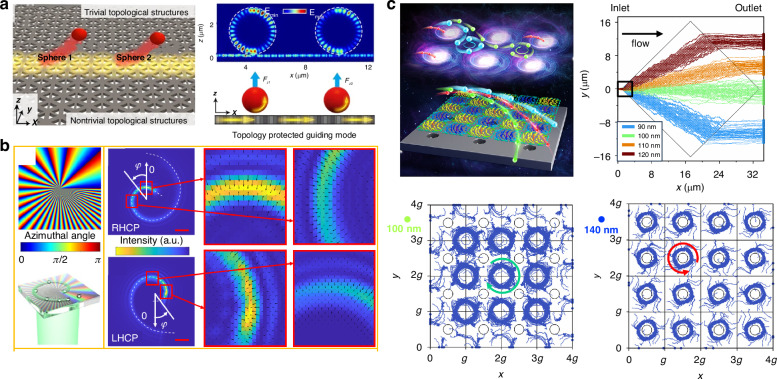
Optical sorting in topological optical fields. **a** Topological optical waveguide for the selective trapping and repelling of particles depending on the particle resonance with the light frequency. Reproduced with permission^457^. Copyright 2022, Chinese Laser Press. **b** Optical sorting of 2 µm and 2.5 µm particles in vortex beams with different topological charges. Reproduced with permission^459^. Copyright 2023, Optica Publishing Group. **c** Optical meron-antimeron lattice for the fast sorting sub-100-nm gold nanoparticles using OLFs. Reproduced with permission^131^. Copyright 2023, American Chemical Society

Recently, topological optical fields have started to showcase some strengths in optical sorting due to their unique light properties that may induce some exotic optical forces. Shi et al. proposed a topological photonic structure, which can serve as a topological optical waveguide, to conquer previous light perturbation and weakness effect in the presence of a particle^457^, as shown in Fig. 21a. Particles that resonate with the frequency of the incident light are repelled, whereas particles of other sizes are attracted. Various particles experience unique two-dimensional optical forces within the topological photonic slab, causing them to move in different directions and facilitating efficient sorting.

Vortex beams with different topological charges can be utilized for optical sorting^314,454,458^. Figure 21b shows an optical sling generated by a liquid crystal optical element^459^. The facile design incorporates two independently controlled geometric-phase liquid crystal optical elements to create two electrically switchable transporting optical slings, enabling the sorting of 2 µm and 2.5 µm particles.

For sorting nanoparticles with a high-precision, very recently, Lu et al. configured a meron-antimeron lattice in a photonic crystal slab^131^, as shown in Fig. 21c. The meron/antimeron^460–464^ is a unique topological texture with a skyrmion number of ±1/2. Nanoparticles with different sizes, e.g., 100 and 140 nm, have different dynamics inside the optical meron-antimeron lattice because of distinct optical forces on them. In the absence of flow, various nanoparticles may be trapped or rotated along different orbits and directions by various optical forces or torques^465^. Upon the application of a flow, they follow distinct trajectories and can be automatically and passively separated with 100% efficiency under appropriate flow velocity and laser power conditions. Notably, in this theoretical work, the simulated optical topological field exhibits a working area of 23 × 23 µm^2^. By enlarging the working area, which can be easily accomplished using the EBL lithography, the sorting efficiency can be further ensured since separation distances of distinct nanoparticles increase linearly with the dimension of the sorting area. The enlarged separation distance, e.g., hundreds of micrometres, can eliminate the influence of the Brownian motion of nanoparticles. Potential experiments can be conducted by designing various outlets using microfluidic technology, and distinct particles can flow into those outlets. When particles are several hundred nanometers in size and observable in a calibrated imaging system equipped with a CCD or EMCCD, their dynamics can be monitored in real time using AI-assisted imaging recognition algorithms, enabling the evaluation of the sorting efficiency. When particles are of similar sizes or below 200 nm, making them difficult to monitor clearly, fluorescence staining can be employed to distinguish them in real time. Alternatively, sorting efficiency can be assessed by quantifying the percentage of target particles collected at the outlet. In this case, the size and type of nanoparticles can be identified through observation of the SEM image or by analyzing different fluorescent emissions. Theoretically, this method can separate 99 nm and 100 nm gold nanoparticles, achieving a remarkable resolution of 1 nm. This work showcases the immense potential of topological optical forces in various biomedical and chemical applications.

Topological optical fields exhibit distinctive phases, optical spins, and confined light intensities, offering numerous opportunities for ultrahigh-precision optical manipulation tasks like sorting, binding, trapping, and transporting.

### Optical sorting assisted by other sorting techniques

Optical sorting sometimes faces the limitation of relatively lower speed compared to other techniques such as microfluidics and acoustics^168,466,467^. However, it holds an advantage in effectively handling nanoscale particles. Combining optical sorting with other sorting techniques may present new opportunities for rapid sorting and sensing of bioparticles^105^.

**Fig. 22:**
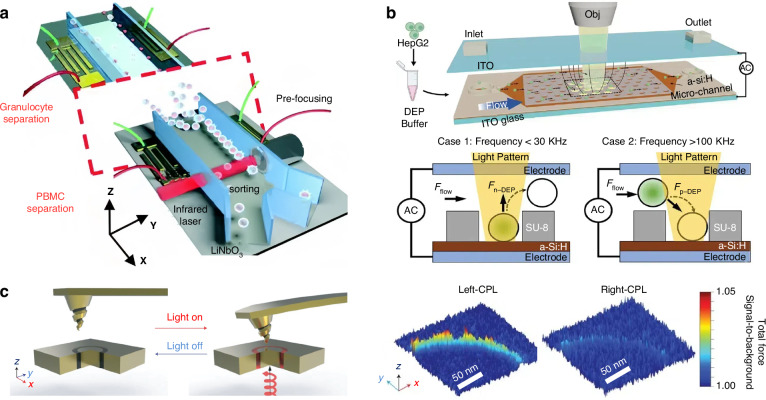
Combination of optical sorting with other sorting techniques **a** High-speed sorting of leukocyte subpopulation integrated acoustic and optical sorter. Reproduced with permission^473^. Copyright 2018, Royal Society of Chemistry. **b** Optoelectronic tweezers micro-well system for highly efficient single-cell sorting. Reproduced with permission^478^. Copyright 2024, Wiley-VCH GmbH. **c** Enantioselective optical forces from a nano-chiral aperture in an AFM configuration^359^. Reproduced with permission^359^. Copyright 2017, Springer Nature

An acoustic sorter is a type of sorting device that utilizes surface or bulk acoustic waves to manipulate particles or cells^468–470^. These sorters typically employ acoustic standing wave fields or acoustic streaming effects to separate particles based on their size, density, or other physical properties. Acoustic sorters can be efficiently used in various applications, including cell sorting, exosome screening and disease dignosis^466,467^. Acoustic tweezers exhibit merits in the high speed, reaching up to 100 µL min^−1^ and label free, and are feasible to handle sub-µm particles^471^. Combining acoustic sorting with optical forces could enable more dynamic manipulation of a wider variety of nanoparticles at high speeds^472,473^. For instance, Hu et al. designed an acoustic-optical chip for the label-free leukocyte subpopulation seperation^473^, as shown in Fig. 22a. They cascaded acoustic and optical sorters to eliminate granulocytes using standing surface acoustic wave, and sort lymphocytes and monocytes that have overlap in size and density using the ORP. In the experiment, a high-throughput sorting was achieved, resulting in 99% purity of lymphocytes, 98% purity of monocytes, and 95% purity of granulocytes, representing a powerful tool for adjunctive clinical diagnosis.

Optoelectronic tweezers are exemplary in combining optical fields with dielectrophoretic forces^389,474–476^. They utilize projected optical images to excite electric fields and generate forces, showcasing advantages in manipulating nano-objects with minimal light and electric powers. In 2005, Chiou et al. proposed an optoelectronic sorting strategy driven by optical images to achieve simultaneous high-resolution and high-throughput sorting of particles^477^. The trajectories of particles can be dynamically controlled by programming the optical imaging projected onto the electrode. By doing so, particles can be massively and effectively trapped, transported, and sorted. This technique can parallelly manipulate 15,000 particles on a 1.3 × 1.0 mm^2^ area using a minimal optical intensity, which is significantly lower than that required by conventional optical tweezers.

Recently, Gan et al. developed an optoelectronic tweezers micro-well system^478^ to sort cells at a high flow rate of 20 µL min^−1^, as shown in Fig. 22b. In this system, cells can be trapped and confined in individual microwells, achieving a trapping rate exceeding 91.9%. By controlling the frequency and power of the applied signal, HepG2 cells can be trapped in the microwells, while L-O2 cells are released. Subsequently, HepG2 cells can also be released from microwells, demonstrating the great versatility of this chip in handling and analyzing single cells. Optoelectronic tweezers can now be employed to manipulate nano-objects such as metallic nanowires and nanospheres^479–481^. However, they typically have limited spatial resolutions constrained by the diffraction limit^389^, which may pose barriers in sorting nanoparticles.

To achieve the nanometer-precision force mapping, Zhao et al. developed a chiral atomic force microscope (AFM) probe coupled with a plasmonic optical tweezer^359^, as shown in Fig. 22c. The optical force on the nanotip depends on both the handedness of the tip and the circular polarization of the incident light. Particularly, transverse forces on a left-handed chiral tip are attractive with left-handed circularly polarized lightwaves and repulsive with right-handed ones. This demonstrates great potential for sorting nano-chiral objects with different handedness. Furthermore, the disparities in total force between specimens of opposite handedness exceed 10 pN. The microscope tip can accurately map chiral forces with a lateral resolution of 2 nm, unveiling a distinct spatial distribution of forces for each handedness. The AFM probe can also be applied in other enantioselective systems, such as the evanescent wave^87,482^, where the OLF arising from the transverse spin can be utilized to sort chiral particles with different handedness.

Another typical particle sorting technique is called deterministic lateral displacement (DLD)^483–486^, which can be utilized to sort a wide range of particles, from exosomes to microparticles. The DLD technique can combine with the magnetophoresis to separate three different particles. For instance, DLD can be employed to eliminate nucleated cells from blood samples. Subsequently, magnetophoresis can be utilized to separate circulating tumour cells from white blood cells^486,487^. By substituting magnetophoresis with an optical sorter, similar performance can be expected to be achieved.

So far, there have been only a few demonstrations of cascaded sorters, while they have great potential in handling complex biosamples that cannot be effectively processed using only one type of sorting technique.

## Conclusions and perspectives

In summary, the theory and techniques of optical tweezers are advancing the manipulation and sorting of particles, with important applications in areas such as single-cell analysis, disease monitoring, clinical diagnostics, microfluidics, and nanotechnology. This review delves into the application of optical tweezers for sorting particles of diverse characteristics. It covers the underlying physics of optical forces utilized for particle sorting and explores the recent progress in sorting technologies and platforms. While optical sorting offers significant benefits like high precision, selectivity, and non-contact operation, it also encounters challenges such as limitations in sorting speed and difficulties in handling high-concentration samples. Anticipated developments in optical sorting involve enhancements towards faster sorting speeds, handling smaller particle sizes, and accommodating larger throughputs. In the future, it is anticipated that the introduction of new materials for light modulation and advancements in light source technology will lead to more effective solutions for optical sorting.

We further summarize and compare the sorting speed, throughput, accuracy and limitations of various optical sorting techniques in Table 2. The speed consists of two components: the image processing speed and the particle moving speed. For commonly used CCD cameras, the frame rate typically ranges from tens to hundreds of frames per second. For specific applications, such as investigating particle transient speeds^133,306,488^ and classifying particles in flow cytometry^489^, high-speed cameras or detectors can be employed. A wide range of experiments are conducted in a static aqueous environment, resulting in low throughput that limits their application to single-particle studies. Optofluidic chips that combine optical forces with microfluidics have great potential to bridge the gap between laboratory studies and clinical applications. However, a single optofluidic chip can be relatively costly due to the intricately designed microchannels and optical structures. Sometimes, nanostructures for sorting nanoparticles require high-cost nanofabrication techniques, such as EBL, which also tend to have a relatively low yield. FACS machines integrated with optical tweezers have capabilities for high-speed screening of bioparticles. Nevertheless, this technique can now only handle sub-microparticles with the assistance of fluorescence labelling^489^. Similar functions can be achieved in microfluidic chips, where particles can also be detected and selectively directed into different outlets based on their distinct signals^167,297,490,491^. The metasurface shows great potential for sorting nanoparticles, although current experimental demonstrations are limited to sub-micrometre sizes^492^. The difficulties arise from the uncompromising requirements for nanofabrication precision, which needs high-precision expensive techniques, such as the EBL and focused ion beam (FIB)^493^. Some methods, including optical lateral forces, have emerged in recent years^131^, demonstrating unprecedented capabilities for sorting nanoparticles with sub-10-nm precision at speeds exceeding 1000 µm s^−1^. However, much of the research in this relatively new field is theoretical, leaving ample opportunity for future exploration.

**Table 2:** Comparison of the sorting speed, limitation, throughput and accuracy of various techniques using some typical examples

Sorting method	Technology	Particle types & Sizes (*d*)	Speed	Throughput	Accuracy/ resolution	Limitation	Ref.
Active sorting	Algorithm	Au/80 ± 9 nm	Image processing: 200 frames s^−1^ Particle moving velocity: ~10 μm s^−1^	Single nanoparticles, tens of nanoparticles per second	Sub-10 nm	Quasi-static, low throughput, require experienced and trained personal	^65^
	Algorithm	Polystyrene/(0.5–20 μm) shaped microparticles	Image processing: $\gg$2220 frames s^−1^ (since it can handle 2220 particles s^−1^)	96 μl min^−1^ or 10^7^ particle h^−1^	Size: 500 nm; Length-diameter ratio difference: 1	High cost, need fluorescence staining for nanoparticles, require experienced and trained personal	^489^
	Fluorescent labelling	A variety of cells or particles	Image processing: 200 frames s^−1^ Particle moving velocity: 80 μm s^−1^	1.4 cells s^−1^	>89% efficiency for microparticles	Potential particle aggregation, speed limited by the hardware and algorithm, normally applicable for microparticles	^266^
	Raman spectroscopy	A variety of cells or particles	Raman signal: <10 frames per second Particle moving velocity: 150 μm s^−1^	Average 3 minutes for the isolation of each cell	~90% of cells are isolated as a single cell in 0.5 μL of PBS	High cost, low throughput, difficult in integration, require database	^282^
Passive sorting	Optical radiation pressure	Au/60–100 nm & *E*. *coli*	Image processing: 100–200 frames s^−1^ Particle moving velocity: <100 μm s^−1^	Tens of nanoparticles	Sub-10 nm	Single particles, low throughput, high-laser power	^7^
	Optical gradient force	Silica, polymer, microcapsules/several micrometers	Image processing: 20–200 frames s^−1^ Particle moving velocity: 20–100 μm s^−1^	25 particles per second	~100% efficiency for deflecting different microparticles	Need specially designed optical pattern, normally only applicable for microparticles	^325^
	Phase-gradient force	Au/80 nm & 100 nm (experimental results)	Image processing: 100–200 frames s^−1^ Particle moving velocity: ~10 μm s^−1^	Single nanoparticles, tens of nanoparticles per second	Sub-10 nm	Quasi-static, low throughput, require experienced and trained personal	^63^
	Optical pulling force	Polystyrene/600–2000 nm	Particle moving velocity: <100 μm s^−1^	Tens of nanoparticles	Up to 90% separation efficiency for sub-100 nm particles	Low speed, applicable for specific sizes, normally require modelling before the experiment	^66^
	Optical lateral force	cholesteric liquid crystal/600–2000 nm	Imaging processing: 100–200 frames s^−1^ Particle moving velocity: ~10 μm s^−1^	Tens of nanoparticles	Depending on the chirality parameters of particles	Low speed, high power	^72^
	Potential well	Polystyrene/100–500 nm	Image processing: 100–200 frames s^−1^ Particle moving velocity: 6–22 μm s^−1^	Hundreds of nanoparticles at a time. More particles can be trapped after releasing previous ones	Trapping efficiency can reach 100%, sorting efficiency >90%, sorting resolution ≤100 nm	Relatively low sorting speed, trained personal in silicon photonics	^362^
	Holographic technique	Silica/1 µm and 1.9 µm	Sorting process requires reassembling particles in ~1 min	Up to hundreds of single particles	94% sorting accuracy	Static sorting, low throughput.	^327^
	Optical field movement	Polystyrene/100–198 nm	Image processing: 60 frames s^−1^ Particle moving velocity: ~10 μm s^−1^	Single nanoparticles	-	Require laser scanning process, suitable for specific particles	^381^
	Optical field enhancement	A variety of cells or particles with size ≤ 100 nm	Image processing: 100–200 frames s^−1^ Particle moving velocity: 50–200 μm s^−1^	Up to hundreds of nanoparticles	>90% sorting efficiency, sorting resolution: ≤10 nm	Require precise nanofabrication process	^59^
	Metasurface	Au/0.3–1.5 µm	-	Selective trapping tens of nanoparticles	-	Moderate beam quality, currently only applicable for microparticles	^492^

The complexities and costs of the setup are significant factors to consider when addressing specific applications, as summarized in Table 3. A typical system usually includes a camera or photon detector, along with a key component for introducing the light field. They are typically the primary costs associated with the setup. For detecting microparticles, a standard CCD camera priced at several thousand USD can be used. However, to detect nanoparticles and nanosized bioparticles, such as sub-100-nm viruses, a highly sensitive EMCCD with dark-field or fluorescence configurations can be used, which typically costs tens of thousands of USD^59^. Some techniques, such as holographic and vector-beam optical tweezers, utilize phase-shaping optical elements like the SLM to create large-field special beams. When combined with the necessary optical systems, these setups may cost tens to hundreds of thousands of USD, depending on the functionalities of different systems. To achieve both the high speed and low cost of sorting nanoparticles with sizes smaller than 200 nm, future efforts can be devoted to designing all-dielectric nanostructures for special light fields, which can generate huge optical forces. In these cases, the primary cost arises from nanofabrication, which can be reduced as nanotechnology advances and the quantity of chips increases.

**Table 3:** Comparison of setup complexities of various active and passive sorting technologies

Sorting method	Technology	Particle types & Sizes (*d*)	Laser wavelength (*λ*)	Laser intensity/power	Key optical components	Imaging/Signal acquiring system	Cost estimation (USD)	Ref.
Active sorting	Algorithm	Au/80 ± 9 nm	800 nm	200–400 mW	SLM	Dark field, CMOS camera	100–250 K	^65^
	Algorithm	Polystyrene/(0.5–20 μm) shaped microparticles	488 nm	Default (for scattering signal)	FACS instrument	photodetector	300–500 K	^489^
	Fluorescent labelling	A variety of cells or particles	1064 nm	67–270 mW	Inverted microscope	CCD camera	100–250 K	^266^
	Raman spectroscopy	A variety of cells or particles	532 nm for Raman excitation; 1064 nm for trapping	13–50 mW	Inverted microscope	Raman spectroscopic CCD	150–250 K	^282^
Passive sorting	Optical radiation pressure	Au/60–100 nm & *E*. *coli*	532 nm	100–500 mW	Inverted microscope	CCD camera	30–50 K	^7^
	Optical gradient force	Silica, polymer, microcapsules/several micrometers	1070 nm	530 mW	Diffractive optical element/SLM	CCD camera	50–300 K	^325^
	Phase-gradient force	Au/80 nm & 100 nm (experimental results)	800 nm	125–400 mW	SLM	Dark field, CMOS camera	100–250 K	^63^
	Optical pulling force	Polystyrene/600–2000 nm	532 nm	3.2 W	Interference system	CCD camera	20–50 K	^66^
	Optical lateral force	cholesteric liquid crystal/600–2000 nm	532 nm	1–1.6 W	Microscope	CCD camera	30–50 K	^72^
	Potential well	Polystyrene/100–500 nm	1550 nm (silicon photonics)	0.43–1.5 mW (output power from the fiber)	Nano-alignment stage	CCD camera	100–200 K	^362^
	Holographic technique	Silica/1 µm & 1.9 µm	532 nm	<5 W	SLM	CCD camera	50–300 K	^327^
	Optical field movement	Polystyrene/100–198 nm	1064 nm	0.8 W	Acousto-optic deflectors	CCD camera	200–300 K	^381^
	Optical field enhancement	A variety of cells or particles with size ≤100 nm	532 nm	40–200 mW	Vertical microscope	Dark field, EMCCD	150–300 K	^59^
	Metasurface	Au/0.3–1.5 µm	532 nm	30 mW	Metalens	CCD camera	20–50 K	^492^

It is worth noting that the temperature increment in optical manipulation can be significant particularly in plasmonic systems^382,399,494^, even though optical powers in those systems are typically below 10 mW. When the temperature is increased to above the boiling point, air bubbles may emerge, which could be a profound issue in trapping and sorting systems^495–497^. Sometimes, air bubbles can be leveraged for efficient particle manipulation^498–500^, while in most cases, they are considered a problem to be avoided. The temperature increase in dielectric systems can be disregarded, as they only exhibit minimal light absorption. In contrast, it has been manifested that moderate laser powers (e.g., 1 mW µm^−2^) can raise the temperature by less than 10 degrees in plasmonic systems, such as metallic nanoparticles or nanostructures^7,58,384,501^. A few degrees of temperature increase can still harm bioparticles exposed to a light field for an extended period. However, this adverse effect can be mitigated when the particles are sorted in a flow stream. Ultimately, using all-dielectric materials for manipulating bioparticles is an optimal choice. Intriguingly, some effects in all-dielectric nanostructures can employed for high-efficiency trapping and sorting. Examples include the BIC^59,407,409^, Fano resonance^60,405,502^, topological features^131,455^ and multipoles^81,503,504^, all of which merit further explorations.

**Fig. 23:**
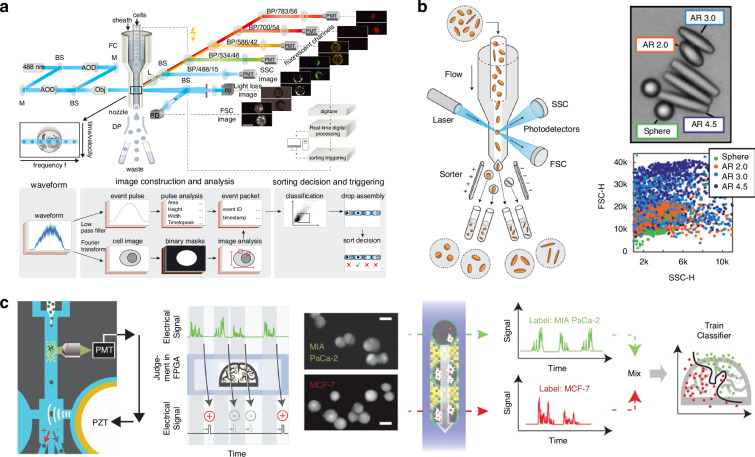
Potential rapid and high-throughput optical sorting through the integration of flow cytometry and artificial intelligence algorithms. **a** Fast and selective sorting of single cells with unique spatial and morphological traits using an AI-enabled sorter. Reproduced with permission^505^. Copyright 2022, American Association for the Advancement of Science. **b** High-speed sorting of microparticles with diverse shapes utilizing a fluorescence-activated cell sorting instrument and imaging analysis algorithm. Reproduced with permission^489^. Copyright 2018, Springer Nature. **c** Sorting of morphologically similar MCF-7 and MIA PaCa-2 cells using the “ghost cytometry”. Reproduced with permission^506^. Copyright 2018, American Association for the Advancement of Science

On the other hand, there exists considerable potential for integrating optical tweezers with AI in the realm of cell sorting. By harnessing focused laser beams for accurate cell manipulation and leveraging the proficiency of AI algorithms in analysing intricate data patterns, supplemented by electronic technologies, this integration holds the potential for automated and accessible cell sorting processes in non-specialized laboratories. For example, a high-speed fluorescent image sorting technology was developed by integrating rapid fluorescence imaging, conventional cuvette-based droplet-sorter, and low-latency signalling electronics^505^. Combining optical detection techniques and real-time sorting electronics, this system enables single-cell sorting based on whole-genome images within a mere nine hours (Fig. 23a). Through the integration of the fluorescence-activated-cell-sorting instrument and image recognition programs, Mage et al. were able to capture scattering characteristics, enabling the categorization of distinct shapes and facilitating shape-oriented particle sorting (Fig. 23b)^489^. Another noteworthy case involves Ota et al., who utilized the SVM algorithm to identify the compressed signal of fluorescent cells measured by a single-pixel detector, thereby eliminating the need for image reconstruction^506^. This technique, known as the “ghost cytometry”^506–508^, demonstrates remarkable accuracy and high throughput, enabling the selective sorting of morphologically similar MCF-7 and MIA PaCa-2 cells at an impressive rate of 3000 cells per second (Fig. 23c).

AI algorithms can be trained to recognize specific cell types based on optical characteristics, such as size and shape^509–511^. When combined with the individual cell manipulation capabilities of optical tweezers, they could enable real-time and high-speed sorting^194,505,512,513^. This integration will facilitate high-throughput screening^514,515^, single-cell analysis^516^, and adaptive sorting strategies^517^, which are promising in fields like drug discovery and disease diagnosis. Additionally, the combination offers enhanced precision and control over cell manipulation, promising advancements in understanding cellular heterogeneity and dynamics, thus paving the way for the future of biomedicine and biotechnology.

In addition to machine learning-enabled optical sorting techniques, utilizing topological optical fields with fascinating energy flux distributions, optical spins, and phases could enhance the capabilities of optical manipulation. For instance, optical sorting using optical meron textures^131^ can achieve a sub-10 nm resolution sorting of gold nanoparticles at a high speed. Sorting using enhanced light-matter interactions, such as multipoles^440^, can further advance the sorting resolution of dielectric particles to 1 nm. Noteworthy, integrated optical sorting could also be possibly realized with recently reported vectorial liquid-crystal holography^518^. Another realm that deserves more exploration is the optical sorting of nano-sized chiral enantiomers, given considerable efforts dedicated to a microscopic scale. This will contribute substantially to biochemical societies^519–521^. Besides optical forces, chiral enantiomers can also be sorted efficiently using optical torques^356,522,523^.

**Fig. 24:**
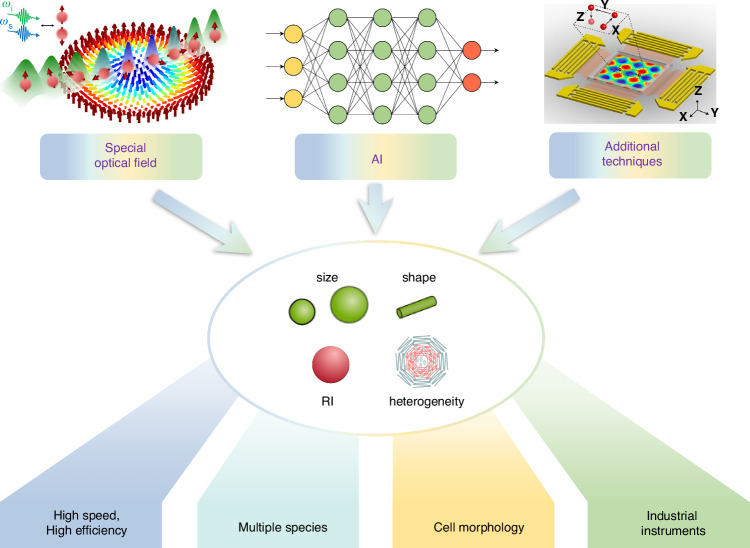
Schematic of the prospect of optical sorting. Special optical field, reproduced with permission^550^. Copyright 2021, Springer Nature; AI, reproduced with permission^551^. Copyright 2020, MDPI; Additional techniques, reproduced with permission^552^. Copyright 2016, National Academy of Sciences; size, shape, RI and heterogeneity, reproduced with permission^553^. Copyright 2020, Springer Nature

Optical sorting is highly promising, with ongoing advancements in technology and increased demands across various industrial implementations. This technique demonstrates unprecedented capability in handling various types of particles with varying sizes, shapes, refractive indices, biological heterogeneity, and other properties (Fig. 24). Compared with other sorting techniques such as acoustics, electrophoresis, membrane filtration, immunoaffinity and microfluidics, one of the greatest advantages of optical sorting is its high precision, as shown in Table 4. Nanoparticles smaller than 100 nm can be separated with sub-10-nm precision using various optical sorting methods^7,63,297,440^. Meanwhile, optical forces can be employed to sort various types of particles, including those made from different materials^297,422^, core-shell particles^35,36,73^, chiral particles^72,361^, particles of various shapes^372,489^, among others. Particles of similar sizes are often challenging to sort using other techniques. However, most optical sorting methods, particularly for nanoparticles, tend to have relatively low throughput. Some optical methods can only manipulate single particles or have a flow rate of less than 1 µl min^−1^.

**Table 4:** Comparison of optical sorting and other sorting methods

Technology		Principle	Particle types & Sizes (*d*)	Throughput	Advantage	Limitation	Ref.
Optical sorting	Conventional optical forces	Size/material/shape-dependent ORP and OGF	A variety of nanoparticles (sub-10 nm) and bioparticles (sub-100 nm)	Single particles or < 10 μl min^−1^	Nanosize, non-invasive,	Low throughput, high laser power	^7,297,362^
	Exotic optical forces	Size/material/shape-dependent ORP and OGF	A variety of microparticles including chiral particles	Single particles (experiment), or < 1 μl min^−1^ (theory)	High precision, applicable for chiral particles	Low throughput, high laser power, for specific particles	^66,128,131^
	Algorithms	Distinguish particles through algorithms	A variety of particles	Single nanoparticles, or tens of nanoparticles per second	High specificity and purity	Complex setup, require experienced and trained personal,	^65,489^
	Fluorescence labelling	Sorting by tagging particles	A variety of bioparticles and microparticles	Up to thousands of cells s^−1^	High specificity and purity	Normally applicable for microparticles	^265,266^
Acoustophoresis		Acoustic radiation force	Exosomes, bacteria, blood components, cells, nanoparticles	0.43 μl min^−1^–1 L h^−1^	Biocompatibility, versatile, avoid high shear stress, non-invasive	Heavy and expensive equipment, normally for micro- and sub-micron particles	^466,524–526^
Electrophoresis/Dielectrophoresis		Electrostatic force	A variety of bioparticles and microparticles	1.25–1000 μl min^−1^	Easily achieved, high selectivity and controllability, high throughput	Induce electrothermal flows and joule heating, strong electric field, electrochemical reaction	^527–531^
Magnetophoresis		Magnetic force	Erythrocyte, leukocyte, eVs, bacteria & 5–1000 nm	0.3–1000 μl min^−1^	High throughput, straightforward protocols, low cost, low heat	Time-consuming sample preparation, strong magnetic field	^532–534^
Centrifugation		Centrifugal force generated by rotation	A variety of bioparticles and nanoparticles	10^6^–6 × 10^8^ particles h^−1^	High throughput	Relatively low resolution	^535–538^
Filtration		Membrane pore size	A variety of bioparticles and nanoparticles	100–500 L (m^2^. h.bar)^−^^1^	Efficient separation, simple operation, nanometre precision	Sample blockage, possibility of damage due to shear stress	^539–541^
Immunoaffinity		Capturing by tagging antibodies	Exosomes, EVs & 30–200 nm	0.13–400 μl min^−1^	High specificity and purity	Biolabelling, low yield, time-consuming, high cost	^542–544^
Microfluidics		Hydrodynamic approaches	A variety of bioparticles and microparticles	0.1 nl–10 ml min^−1^	Simple, easy to operate, relatively cheap	Relatively low resolution, normally for microparticles	^545–547^

Further efforts encompass designing specialized functional light fields, advancing AI algorithms, and integrating auxiliary sorting techniques, such as acoustics and microfluidics. These endeavours aim to push the limits of current optical sorting approaches, achieving faster and more efficient strategies for multiple species sorting and studying cell morphology.

It can be envisioned that an increasing number of prototypes and instruments for optical sorting with nanometre precision will emerge and be widely deployed in institutes and hospitals, facilitating processing a broad range of chemical particles and biosamples, such as pharmaceutical molecules, viruses and exosomes.
